# Therapeutic Lymphaticovenular Anastomosis for Breast Cancer-Related Lymphedema: A Critical Narrative Review of Comparative Evidence

**DOI:** 10.3390/jcm15207909

**Published:** 2026-10-13

**Authors:** Stiven Yusufov, Evgeniia Iliina, Maria Ermoshchenkova, Igor Reshetov, Olesya Starceva

**Affiliations:** Department of Oncology, Radiotherapy and Reconstructive Surgery, N.V. Sklifosovsky Institute of Clinical Medicine, Sechenov First Moscow State Medical University ( Sechenov University), Moscow 119991, Russia

**Keywords:** BCRL, LVA, ICG lymphography, lymphedema

## Abstract

This critical narrative review examines clinical outcomes of therapeutic lymphaticovenular anastomosis (LVA) for established breast cancer-related lymphedema and proposes a coordinated outcome assessment. MEDLINE/PubMed, Embase, Scopus, the Cochrane Library, and ClinicalTrials.gov were searched through 6 September 2026. Comparative studies were selected purposively, with clinical series, imaging and physiological studies, and evidence syntheses providing context. We examined outcomes, comparators, follow-up, and study limitations without quantitative pooling. A six-month interim randomized analysis found no convincing between-group differences in the total Lymphedema Functioning, Disability and Health (Lymph-ICF) score or volume measures. The physical domain and reported compression use favored LVA plus conservative treatment. These findings require consideration of outcome hierarchy and multiple comparisons. Selected observational studies reported improvements but were vulnerable to confounding. The proposed framework coordinates a minimum clinical dataset, including prescribed and actual compression use, with optional measurements of anastomosis status, transport, and tissue characteristics. It aligns technical and clinical assessments over time to explore their relationship. The selected clinical findings suggest possible benefits in symptoms and treatment burden but do not establish sustained independence from compression. The framework is a research proposal and does not provide validated rules for patient selection.

## 1. Introduction

Upper extremity lymphedema after breast cancer treatment is a heterogeneous chronic condition in which impaired lymphatic transport coexists with inflammatory and fibroadipose tissue changes. The same increase in limb volume may therefore be accompanied by different degrees of heaviness, pain, functional limitation, and need for compression therapy. Volume remains an important objective outcome, but it does not fully characterize the clinical condition [1,2].

Compression therapy, physical activity, skin care, and other individually prescribed components of conservative treatment form the basis of BCRL management [3,4]. LVA creates an additional connection between a lymphatic collector and a recipient venule, allowing lymph to bypass an area of impaired regional transport. The procedure does not restore removed lymph nodes or the original anatomy of regional pathways, and outcomes remain dependent on concomitant treatment. Collector status, venous hemodynamics, the technical construction of the anastomosis, and tissue phenotype are considered possible determinants of response, but their independent contributions to clinical benefit in BCRL remain poorly defined.

The comparative evidence comprises studies with different control strategies, follow-up durations, and endpoints. In the six-month randomized comparison by Jonis et al., there was no convincing difference in total Lymph-ICF scores or volume measures, whereas the physical domain and compression garment use favored the surgical group [5]. Nonrandomized comparisons report more favorable changes in selected outcomes after LVA but remain vulnerable to confounding by indication and differences in concomitant treatment [6,7]. These findings should be compared in relation to the specific clinical question rather than set against one another solely on the basis of study design.

Existing reviews already consider outcomes of lymphatic surgery beyond volume reduction and discuss the choice among LVA, VLNT, and reductive procedures [8,9]. Recommendations have also been published for core outcome domains and measurement instruments in BCRL and for a minimum set of measures in lymphatic surgery [10,11]. This critical narrative review examines the clinical outcomes reported in selected comparative studies of therapeutic LVA for established BCRL and the main challenges in interpreting them. We use these challenges to propose a framework for coordinated assessment of technical and clinical outcomes, with explicit recording of compression exposure. The framework is intended to complement existing outcome recommendations by aligning these assessments over time.

## 2. Materials and Methods

### 2.1. Study Design and Literature Search

This study was conducted as a critical narrative review. Its focus is therapeutic LVA for established BCRL. Preventive procedures performed during lymph node dissection are considered only to distinguish the research questions and are not used as direct evidence of efficacy in treating established lymphedema.

Publications were selected purposively for their relevance to the clinical comparisons and outcome interpretation addressed in this review. Supporting studies were used to clarify measurement issues and inform the proposed assessment framework. MEDLINE/PubMed, Embase, Scopus, the Cochrane Library, and ClinicalTrials.gov were searched through 6 September 2026. Reference lists of key publications were also reviewed. English-language studies were considered.

The selection process was not intended to identify all literature exhaustively. Exact search strings were not retained, and study selection and data extraction were not performed independently in duplicate. All statements about gaps in the evidence therefore apply only to the publications included in this review. The supplementary tables (Tables S1 and S2) likewise describe this purposively assembled body of literature and do not provide an exhaustive evidence map. When published information is unavailable, we distinguish three situations. A measure was explicitly not obtained, its result was not reported, or the publication does not make clear whether it was obtained.

### 2.2. Organization of the Evidence

The evidence is organized into four areas. Comparative clinical studies are considered separately according to whether they examine the addition of LVA to conservative treatment, compare LVA with other operations, or compare technical strategies. Studies of other anatomical sites provide indirect evidence for BCRL. Uncontrolled series are used to describe feasibility, adverse events, postoperative changes, and prognostic associations. Imaging studies are assessed in relation to their specific purpose, such as mapping, tissue characterization, or evaluation of status and transport at the anastomosis. Experimental and physiological studies inform discussion of biological plausibility but do not establish clinical efficacy.

Systematic reviews, guidelines, and consensus statements are used to compare interpretations and provide clinical context. Their conclusions are not treated as independent confirmations of findings from primary studies already included in this review.

### 2.3. Principles of Interpretation

For each comparative study, we distinguish the population, treatment strategies, outcome, assessment time point, and type of contrast analyzed. Randomized and nonrandomized studies may address the same clinical question but differ in their resistance to bias. Within-group change, between-group differences, and prognostic associations are not treated as interchangeable findings.

For comparative studies, we described specific features of design and reporting that could affect interpretation. These included group formation and potential confounding, deviations from assigned treatment, completeness of follow-up, outcome measurements, and completeness of outcome reporting. We did not formally assess risk of bias using RoB 2 or ROBINS-I or assess certainty of evidence using GRADE. Standardized risk-of-bias categories were therefore not assigned. The tables present observed features of each study and their possible implications for interpretation without an overall author-assigned risk-of-bias rating.

A shared center or research group is treated as a reason to investigate possible cohort overlap, not as evidence that samples are dependent. Confirmed overlap, probable overlap, and undetermined status are described separately. Reports from the same cohort are considered together and are not treated as independent studies. We likewise distinguish an absence of measurement, absence of published measurement results, and insufficiently clear reporting.

## 3. Comparative Clinical Evidence

Comparative studies of LVA differ in their populations, control strategies, surgical techniques, assessment times, and outcome definitions. Before comparing findings, it is necessary to determine whether the studies address the same clinical question. Randomization reduces the influence of baseline confounding. Nonrandomized comparisons require additional assumptions and appropriate adjustment. Before-and-after series describe changes following an intervention but do not establish what would have happened without it. The studies below are considered with these distinctions in mind. The principal comparative studies in BCRL are summarized in Table 1.

### 3.1. Randomized Comparative Studies

Jonis et al. randomized 100 women with unilateral BCRL and ICG-visible lymphatic vessels to LVA plus conservative treatment or conservative treatment alone. The interim analysis included 92 participants who had reached six months of follow-up (46 per group). Of these, 90 had early-stage II disease, labeled “IIa” by the authors [5]. Further study characteristics and reporting details are provided in Table S1.

At six months, the between-group difference in total Lymph-ICF score was −5.92 points (95% CI −14.88 to 3.04). There were no convincing differences in the water displacement volume measure or UEL index. The physical domain favored LVA (reported difference −13.77 points; *p* = 0.001). In the LVA group, 10 patients reported complete and another 10 partial discontinuations of compression garments, compared with none in the control group [5]. These findings suggest possible benefits in physical symptoms and treatment burden. The domain result should be interpreted alongside the total score, given the open-label interim design and multiple comparisons. Nonsignificant differences do not establish an absence of benefit, and reported compression changes do not establish sustained independence.

Soft tissue infections were reported as adverse events, without a formal between-group analysis of infection episodes [5].

Kleeven et al. evaluated cost effectiveness over two years in the same randomized cohort. The adjusted difference was 0.045 quality-adjusted life-years (QALYs) in favor of LVA (95% CI −0.021 to 0.112). At a willingness-to-pay threshold of €20,000 per QALY, the estimated probability of cost effectiveness was 0.60 from a societal perspective and 0.08 from a healthcare system perspective. The authors described more favorable clinical outcomes and reduced compression use, but deferred detailed results to a separate report [14]. This economic analysis does not replace detailed long-term clinical effectiveness data.

Mihara et al. compared LVA plus decongestive therapy with decongestive therapy alone in a randomized trial of secondary lower-limb lymphedema. The primary analysis included 225 of 336 randomized participants. The LVA group had a greater reduction in cellulitis episodes recorded over six-month periods (between-group difference in change −0.35; 95% CI −0.62 to −0.09). Secondary assessments in 170 participants favored LVA for palpated firmness at selected thigh sites, but not convincingly for circumference or pain. Missing outcome data, deviations from assigned treatment, and differences in concomitant therapy limit the interpretation. These findings provide indirect evidence for BCRL [15].

In their randomized pilot trial, van Mulken et al. compared robot-assisted with manual LVA in patients with BCRL [16]. A subsequent report presented one-year outcomes [17]. The comparison concerns operative techniques and feasibility rather than the added benefit of LVA over conservative treatment.

### 3.2. Nonrandomized Comparative Studies

Shimbo et al. retrospectively compared LVA plus compression therapy (29 patients) with continued conservative treatment (52 patients) in early BCRL. UEL index changes favored LVA at assessments from 6 to 24 months (all *p* < 0.001). This index is based on arm circumferences and BMI; its percentage change is not equivalent to percentage limb-volume reduction. Cellulitis was reported in 0/29 and 8/52 patients, respectively, with mean follow-ups of 32.9 and 51.2 months [6]. These findings suggest possible benefit, although nonrandomized treatment selection limits causal interpretation and unequal follow-up complicates the infection comparison.

Seidenstuecker et al. retrospectively compared LVA (47 patients) with vascularized lymph node transfer (VLNT; 58 patients) in chronic BCRL. A two-year follow-up was available for 96 patients. Both groups showed reduced arm circumference, without a convincing between-group difference in the reported relative reduction measure (*p* = 0.711). Eleven postoperative complications, mostly minor, were reported after VLNT, compared with none after LVA. Nonrandomized treatment selection and larger baseline arm circumferences in the VLNT group limit a comparison of the procedures [12].

Kappos et al. retrospectively compared LVA (42 patients) with VLNT (70 patients) in chronic BCRL. A one-year follow-up was completed by 107 patients, with outcomes reported up to 24 months. The circumference-based relative reduction peaked at three months after LVA, then declined and stabilized. VLNT showed peaks at three and eighteen months. Nonrandomized treatment selection limits a direct comparison of these patterns [13]. Possible participant overlap with the Seidenstuecker cohort remains unconfirmed [12,13].

In a prospective nonrandomized study of 92 patients with BCRL, Zeltzer et al. compared LVA (30 patients), VLNT (15), and combined surgery (13), each added to complex decongestive therapy (CDT), with CDT alone (34). At one year, the reported mean change in relative interlimb volume difference (RID) favored LVA plus CDT over CDT alone (−6.21% ± 5.76% versus +3.23% ± 6.83%; *p* < 0.0001) [7]. According to the study registry (NCT06606145), control patients had declined surgery, leaving the comparison susceptible to selection bias.

These studies address two distinct comparisons: LVA added to conservative treatment versus conservative treatment alone [6,7] and LVA versus VLNT [12,13].

### 3.3. Other Reports on Clinical Changes, Patency, and Quantitative Measurements of Lymph Flow

Uncontrolled studies provide information on technical feasibility, complications, anastomotic patency, and postoperative clinical changes. These observations alone do not establish the added benefit of LVA over conservative treatment.

Postoperative clinical changes: In a prospective series of 100 cases, Chang et al. reported a mean 42% reduction in interlimb volume difference at 12 months in the upper-limb BCRL subgroup [18]. Qiu et al. prospectively followed 100 patients, including 85 with BCRL. After a mean follow-up of 25 months, the total Lymph-ICF score had decreased by 13.3 points (*p* < 0.001), while the UEL index showed no convincing change. At the last assessment, 43% of the total cohort had completely discontinued compression garments [19].

Longer-term reports provide additional clinical observations. In Thomas et al.’s cohort of 150 patients with upper- or lower-limb lymphedema, quality of life and symptoms improved and compression use decreased over 24 months, without a reduction in mean perometry or circumference measurements [20]. Beederman et al. also reported volume and functional improvements, although their cohort included different limb sites and both LVA and VLNT [21]. Hahn et al. analyzed 92 patients with upper- or lower-limb lymphedema at a mean follow-up of 77 months and reported improved Lymph-ICF scores. Possible overlap with the earlier Qiu cohort remains unconfirmed [19,22]. These observations illustrate the value of assessing limb size, patient-reported outcomes, and compression use together.

Anastomotic patency: Wolfs et al. evaluated 25 patients with BCRL who were also included in the larger Qiu cohort [19,23]. At 12 months, ICG lymphography identified at least one patent anastomosis in 19/25 patients; 34/47 individual anastomoses were classified as patent. Imaging was inconclusive in two patients. Changes in Lymph-ICF, UEL index, and compression use favored patients with patent anastomoses, but between-group differences were not statistically significant. This small exploratory comparison does not establish patency as a surrogate for clinical benefit [23].

Exploratory technical and phenotypic observations: Hashem and Yamamoto used ultrasound mapping for LVA in 23 patients with BCRL and reported a lower UEL index and cellulitis frequency at one year. Greater UEL index improvement was observed in patients with dermal backflow on lymphoscintigraphy. Low adherence to prescribed compression complicates the interpretation [24].

Seki et al. reported a lower UEL index after “dynamic” LVA in 17 patients with ISL stage II BCRL who were unable or unwilling to use compression [25]. Hamada et al. described site-specific circumference changes and discontinuation of different compression garments in 20 patients with secondary upper-limb lymphedema [26]. These observations support documenting the type and extent of compression reduction, but do not establish that compression can routinely be omitted after LVA.

Additional studies explored postoperative measurements: Roh et al. reported correlations between bioelectrical impedance measures and circumference-based limb volume in 25 patients with BCRL assessed before and six months after LVA [27]. Myung et al. described changes in forearm tracer uptake and washout at one year in 18 patients with BCRL using Tc-99m phytate lymphoscintigraphy [28]. These approaches may complement clinical outcome measurements in the optional research module.

Quantitative measurement of lymph flow: Patency alone does not quantify lymph transport. Miseré et al. used transit-time ultrasound flowmetry in 25 patients with peripheral lymphedema. The mean local flow was 0.25 ± 0.19 mL/min before and 0.27 ± 0.22 mL/min after anastomosis creation (*p* = 0.340) [29]. Jonis et al. also measured lymph flow during radial forearm flap reconstruction in 20 patients without a history of limb lymphedema [30]. These findings support technical feasibility, while the relationship between local perioperative flow and subsequent clinical outcomes requires further study.

### 3.4. Systematic Reviews and Meta-Analyses. Pooling Data Does Not Remove the Limitations of Primary Studies

Rosian and Stanak rated the certainty of evidence for LVA effectiveness and safety as very low in their review of prospective studies of primary and secondary lymphedema [31]. Chang et al. supported a possible reduction in lymphedema severity after LVA, but reported no consensus on whether LVA or VLNT was superior [32].

Among the reviews focused on BCRL, Lilja et al. described variable changes in arm volume and improvements in symptoms and quality of life after LVA, while noting that much of the evidence came from studies at a high risk of bias [8]. Zapata-Ospina et al. also reported favorable changes in limb size and quality of life after LVA combined with CDT, based mainly on before-and-after studies [33]. Table S2 summarizes the scope, findings, and principal limitations of the selected reviews.

Brown et al. pooled prospective studies of therapeutic LVA and reported postoperative reductions in limb volume and compression use, together with symptom relief [34]. Kumar et al. reported improved quality of life after therapeutic LVA for established BCRL and considered preventive LVA separately [35]. In interpreting these findings, postoperative changes should be distinguished from between-group estimates of additional benefit.

Broader reviews also examined outcomes beyond limb size. Zurfluh et al. reported improvements in quality of life and fewer cellulitis episodes after lymphatic reconstructive surgery [9]. Meuli et al. found reductions in excess circumference, excess volume, and infection frequency, although their pooled estimates combined LVA and VLNT and therefore describe microsurgical treatment overall [36].

Hahn et al. reported improvements after both LVA and VLNT, but differences in the contributing cohorts and measurement methods limit comparisons between their separate pooled estimates [37]. The network meta-analysis by Zhang and Lodi found no clear difference between LVA and VLNT in therapeutic outcomes, while noting the limited availability of direct comparative evidence [38].

Jungbauer et al. reported fewer annual cellulitis episodes after LVA and VLNT in studies with at least 24 months of follow-up. These findings concern secondary upper- and lower-limb lymphedema and are not restricted to BCRL [39].

### 3.5. Clinical Outcome Domains and Prognostic Features Among Patients Who Underwent Surgery

#### 3.5.1. Outcomes After LVA Belong to Different Clinical Domains and Are Not Interchangeable

Limb volume reflects both fluid and tissue components. Volume measures, circumferences, and the UEL index are not interchangeable; interpretation depends on the calculation method, measurement conditions, and compression use before assessment.

Patient-reported measures assess symptoms, function, and participation in daily life; changes may occur without substantial volume reduction. Open-label assessments may be influenced by treatment expectations. Individual response thresholds and mean between-group differences answer different questions; thresholds require justification for the instrument and population [40,41,42,43]. The original validation publication for the LYMPH-Q Upper Extremity Module did not report such a threshold [44].

Compression use reflects treatment burden and can also influence volume and symptoms. Interpretation depends on prescribed and actual use, changes in the regimen, and resumption after discontinuation. The reviewed series illustrate differences in adherence and the ability or willingness to use compression [24,25].

Infection outcomes depend on event definitions, ascertainment, and follow-up durations. Episode rates and the proportion of affected patients are distinct measures. Changes in conservative care and event recording may contribute to before-and-after differences.

Anastomotic patency, visualized transport, lymphoscintigraphic changes, and quantitative flow describe technical or functional outcomes. The reviewed studies do not validate them as substitutes for patient-important outcomes.

#### 3.5.2. Prognostic Features Among Patients Who Underwent Surgery

Selected reports describe associations of disease stage or lymphatic transport measures with outcomes after LVA [8,18,24,45]. An exploratory analysis by Jonis et al. did not identify clear associations between the characteristics examined and changes in Lymph-ICF [5]. Differences in staging, measurement methods, and the selection of patients for surgery limit interpretation. These reports do not establish which patients benefit more from LVA than from conservative treatment.

### 3.6. Safety and Technical Feasibility of LVA

Intervention safety and the course of the disease are distinct but partly overlapping areas of assessment. Surgical site infection, an episode of cellulitis or erysipelas in the affected limb, and infection at another site should be recorded separately. A single episode may be counted as both a clinical disease event and an adverse event if the protocol provides for this. Interpretation requires information on the site, timing, severity, treatment, and suspected relationship to the intervention.

The level of analysis, follow-up period, and denominator should be prespecified for each event. The number of patients with a complication, the number of recurrent infection episodes, and the number of anastomoses with confirmed patency do not represent the same frequency. Definitions, units of analysis, and reasons for missing data are provided in Table S3.

#### 3.6.1. Surgical Adverse Events

Jonis et al. reported adverse events in both randomized groups, including soft tissue infections [5]. Hashem and Yamamoto reported superficial wound infections in 2/23 patients who were managed with dressing changes [24]. Wolfs et al. reported wound infections in 3/25 patients who were successfully treated with antibiotics [23]. In a retrospective comparison, Seidenstuecker et al. reported no postoperative complications in the LVA group (47 patients) and 11 complications in the VLNT group (58 patients) [12].

An explicit statement that no complications occurred should be distinguished from a lack of safety reporting. Limited details on specific events, such as lymphorrhea or reintervention, may restrict conclusions about their frequency but does not by itself invalidate other reported safety findings.

#### 3.6.2. Technical Feasibility and Failure

Technical reporting should distinguish identification of a suitable vessel pair, anatomical completion of the anastomosis, immediate directional transport, and anastomosis status at follow-up.

Hashem and Yamamoto reported that a lymphatic vessel was not identified at 4/54 sites [24]. Gong et al. calculated technical success as successful anastomoses divided by exploratory incisions [46]. Neither denominator is interchangeable with the number of treated patients.

For future studies, we recommend reporting the unit of analysis (patient, incision, mapped lymphatic vessel, or anastomosis), numerator (*n*), denominator (*N*), and assessment time point for each technical outcome. Follow-up should distinguish confirmed patency or transport, confirmed occlusion, indeterminate findings, examinations not performed, and loss to follow-up. Nonvisualization alone should not be classified as confirmed occlusion. Reports should state how many patients and anastomoses were eligible for assessment and actually examined. Analyses should account for multiple observations within each patient (Table S3).

### 3.7. Generalizability and Translation into Clinical Practice

Generalizability is limited because most studies were conducted in specialized centers by experienced supermicrosurgical teams. A learning curve effect is plausible, but chronological cohorts cannot separate it from concurrent changes in imaging, surgical technique, patient selection, and postoperative management. NICE also notes that lymphatic microsurgery is available in only a limited number of centers [4].

## 4. Why Study Results Differ

Potential sources of variation between studies can be grouped into patient, surgical, and study factors (Table S4). Patient factors include disease stage and duration, lymphatic vessel characteristics, and the relative contributions of fluid accumulation, adipose tissue, and fibrosis.

Surgical factors include mapping methods, the number and configuration of anastomoses, and concomitant procedures. Operative experience and postoperative care may also contribute to variations.

Study factors include the comparator, treatment allocation, outcome definitions, follow-up duration, and documentation of prescribed and actual compression use. The comparator defines the treatment question, while study design and measurement methods influence how the findings can be interpreted and compared.

These factors represent possible explanations for heterogeneity rather than established causes of individual study results. The following biological and imaging sections explain the rationale for additional tissue and transport measurements. Section 8 brings the clinical characteristics, procedural details, compression exposure, and outcomes together in a coordinated assessment schedule, with optional mechanistic measurements selected according to the study question.

## 5. Biological Basis for a Response

### 5.1. What LVA Creates

**Figure 1:**
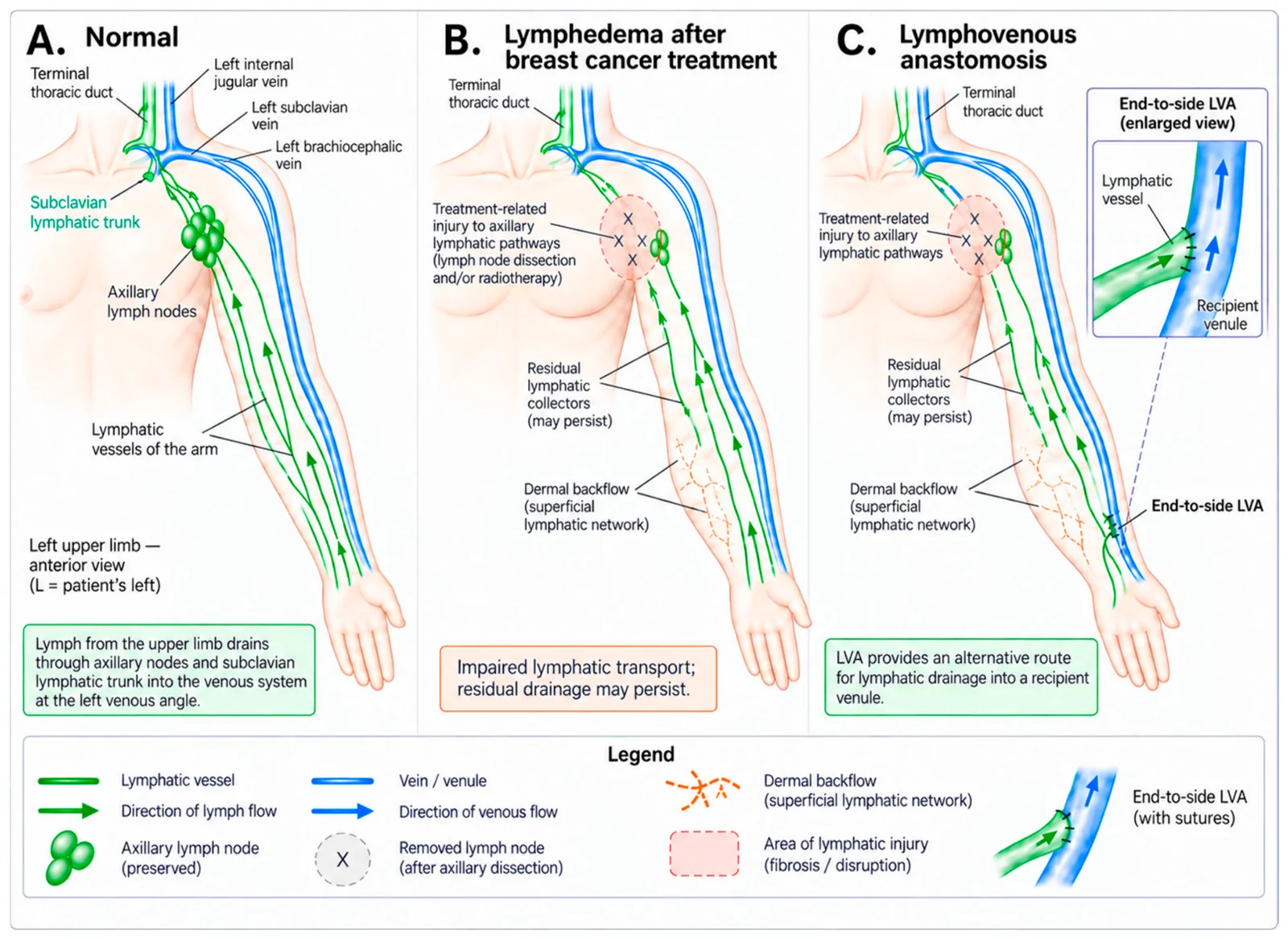
Schematic representation of lymphatic transport and LVA: (**A**) normal regional return, (**B**) impaired transport in BCRL, and (**C**) an additional peripheral connection between a lymphatic collector and a venule. The arrow at the LVA indicates the presumed direction of lymphovenous transport. The diagram does not show vessels to scale, include all collateral pathways, or indicate an established magnitude of additional clearance. The configuration shown is illustrative.

LVA connects a peripheral lymphatic collector to a small vein or venule, creating an additional drainage route without restoring removed lymph nodes or the original regional pathways [5] (Figure 1). Patency does not necessarily imply sustained flow, and contrast passage describes transport under the conditions of an examination [47]. Local flow measurements should also be distinguished from the additional drainage achieved by the limb as a whole [29].

### 5.2. Impaired Lymphatic Transport and Tissue Remodeling in BCRL

In BCRL, damage to regional lymphatic pathways is accompanied by altered drainage and inflammatory and fibroadipose tissue changes [1,2,48,49,50]. These changes help explain why lymphatic transport and tissue composition are considered separately in the proposed framework.

In six women with BCRL, Torrisi et al. reported reductions in selected inflammatory and fibrotic skin changes six months after LVB. The small sample and absence of a parallel group without LVB limit the interpretation of these findings [51].

ICG lymphography maps superficial lymphatic pathways but does not directly quantify fluid, adipose tissue, or fibrosis. ICG stage depends on the classification used, and a visible drainage pathway does not establish normal transport capacity. These findings alone do not establish the expected clinical benefit of LVA [52,53].

### 5.3. The Donor Lymphatic Collector, Intrinsic Contractility, and External Influences on Lymph Flow

Lymph flow depends on intrinsic collector contractility, valve function, pressure gradients, and external mechanical forces [47,54]. Human cuff-based studies have assessed lymphatic pumping pressure, but these measurements do not directly describe donor-collector contractility or flow through LVA [55,56]. The NECST (Normal–Ectasis–Contraction–Sclerosis Type) classification describes collector morphology rather than measured contractility [57].

Movement, limb position, and compression can influence lymph flow. Intraoperative measurements under anesthesia with the limb immobile may not reflect flow during everyday activity. Recording measurement conditions and changes in activity and compression exposure can help interpret longitudinal functional measurements. Prescribed and actual compression use are recorded separately in the proposed framework.

### 5.4. The Recipient Venule, Local Hemodynamics, and Proximal Venous Outflow

Venous pressure and the pressure gradient across an LVA can influence flow direction and persistence. The BSO (backflow, slack, outlet) classification and the subsequently proposed hydropower category describe observed intraoperative venular behavior [58,59]. These observations do not directly measure pressure or establish sustained outflow during everyday activity. Their relationship with later clinical outcomes remains a research question.

Local recipient-venule hemodynamics should be distinguished from proximal venous outflow. CT-detected proximal stenosis does not by itself establish pressure in the recipient venule. In a cohort of patients with secondary lymphedema scheduled for LVA, proximal stenosis was reported more frequently with upper than lower limb involvement [60]. This observation supports further study of venous outflow but does not establish that routine CT venography or treatment of stenosis improves LVA outcomes.

### 5.5. Tissue Substrate and Candidate Fluid-Sensitive Measures

Extracellular water estimates do not describe the full composition of lymphedema fluid. Interstitial fluid in BCRL contains proteins [61]. Inflammatory and fibrotic skin changes have also been documented in BCRL [51]. Water-sensitive measures alone do not capture this biological complexity or identify a reversible fluid fraction.

Bioimpedance spectroscopy (BIS) and its derived L-Dex index provide indirect electrical measures sensitive to extracellular fluid distribution [62]. Pitting reflects tissue displacement under pressure. Extracellular-to-total water ratios depend on both water compartments, while T2-weighted MRI signals depend on tissue characteristics and the imaging protocol. These measures are not interchangeable, and interpretation depends on the anatomical region and measurement conditions.

The MR fat fraction describes relative tissue composition, whereas subcutaneous tissue thickness is a geometric measure that may also change with edema. Shear wave elastography (SWE) measures shear-wave velocity as an indicator of tissue stiffness [63,64]. These measurements should not be interpreted as direct quantification of fibrosis.

## 6. What Each Imaging Method Shows and Which Conclusions It Supports

Imaging methods used in LVA address vessel mapping, tissue characterization, and postoperative assessment. Their use for surgical planning is summarized by van Heumen et al. [65]. Successful mapping does not by itself establish the ability to predict clinical benefit. Table S5 summarizes each method’s signal, intended use, and limits of interpretation.

ICG lymphography visualizes superficial lymphatic pathways and dermal backflow patterns [66,67]. Nonvisualization does not necessarily indicate anatomical absence because detection depends on vessel depth and examination conditions. The classification system should be specified when reporting ICG stage [65,66].

Ultrasound can map lymphatic collectors and recipient venules before LVA. The reviewed reports used high-frequency ultrasound at 18 MHz [24] and ultra-high-frequency ultrasound (UHFUS) at 48–70 MHz [68]. Their mapping results concern vessel detection and intraoperative confirmation of selected targets. Imaging depth and operator experience should be considered when interpreting these technical results.

Contrast-enhanced ultrasound (CEUS) uses intradermally injected microbubbles to map lymphatic pathways and can reveal vessels not visualized by ICG lymphography [69]. A small feasibility study compared microbubble agents in unaffected contralateral limbs of patients with lymphedema [70].

Gong et al. reported shorter anastomosis times and more favorable results for some postoperative measures with CEUS plus ICG than with ICG alone [46]. Tang et al. reported shorter preoperative vessel localization times with CEUS plus ultrasound than with ICG lymphography [71]. The sequential, nonrandomized designs of these two studies limit causal interpretation.

Magnetic resonance lymphangiography (MRL) includes noncontrast and contrast-enhanced techniques. T2-weighted imaging depicts fluid-sensitive signal and tissue morphology, whereas MRL, after interstitial gadolinium injection, visualizes lymphatic anatomy and contrast transport. Protocol differences should be considered when interpreting these findings [72,73].

Li et al. reported the feasibility of combining MRI and CEUS for preoperative lymphatic mapping [74]. Zhu et al. reported close correspondence between vessels mapped using contrast-enhanced MRL with ICG and those located during surgery [75]. These mapping findings should be distinguished from prediction of subsequent clinical benefit.

Postoperative assessment distinguishes anatomical continuity, contrast passage, and sustained transport. In a small case series of lower-limb lymphedema, Suzuki et al. used photoacoustic imaging to obtain three-dimensional images of postoperative LVAs, including visualization of ICG beyond an anastomosis within a vein. Patency could not be assessed in every case [76].

An inconclusive examination should be distinguished from confirmed occlusion. In the proposed framework, anastomosis status, transport measurements, and clinical outcomes are recorded separately (Section 8).

## 7. The Role of LVA in BCRL Treatment

To place the clinical findings in context, Table 2 summarizes selected consensus documents, evidence assessments, and proposed treatment algorithms relevant to therapeutic LVA in BCRL.

The ISL consensus considers microsurgery an adjunct to conservative treatment or an option when conservative treatment has been unsuccessful. Continued compression or physiotherapy may be needed after surgery, particularly in advanced disease [3].

The Lymphedema Summit and Barcelona consensus documents address patient assessment, preoperative preparation, and postoperative care [77,78].

In its 2025 review, NICE noted uncertainty about the effectiveness, safety, and long-term outcomes of surgery for BCRL, as well as the cost effectiveness of LVA in the UK. It nevertheless supported discussing surgical options through shared decision-making after referral to a specialist lymphoedema service [4]. The clinical and economic searches reported in this assessment were conducted in February 2024, before publication of the two-year economic analysis by Kleeven et al. [14].

LVA and VLNT aim to improve lymphatic drainage, whereas liposuction removes excess adipose tissue [8,79]. In a prospective BCRL series, Hoffner et al. reported a sustained reduction in excess arm volume after liposuction with continued compression [80]. Liposuction followed by LVA has also been described in BCRL [81]. These examples highlight the importance of considering the complete treatment sequence and compression exposure when interpreting postoperative outcomes.

Technical suitability for an anastomosis does not by itself show how much benefit LVA adds to conservative treatment. Shared decision-making includes discussion of patient goals, previous conservative treatment, and the possible need for continued compression [3,4,77]. Section 8 therefore proposes a coordinated assessment of technical findings, compression exposure, and patient-important outcomes.

## 8. Priorities for Prospective Research

### 8.1. Questions Already Addressed in Published Protocols

Published protocols address complementary questions. N-LVA specifies a comparison of LVA with sham surgery [82] while the pragmatic LYMPH and SurLym trials assess reconstructive lymphatic surgery added to conservative treatment [83,84]. SurLym also specifies a 36-month follow-up and a planned reduction in compression wear time [84].

A registered trial for lower-limb lymphedema plans clinical outcome assessment alongside anastomotic patency assessment, lymphoscintigraphy, and a skin biopsy substudy [85]. Our proposed framework focuses on coordinating these measurements over time and interpreting them alongside compression exposure.

### 8.2. Core Clinical Measurements and an Additional Mechanistic Module

The proposed framework complements published outcome recommendations for BCRL and lymphatic surgery [10,11]. It separates a minimum clinical dataset from optional mechanistic measurements and coordinates their timing with documentation of prescribed and actual compression use. It is a research proposal, not a new core outcome set or a patient selection rule. Its feasibility and reproducibility remain to be evaluated.

The proposed minimum clinical dataset includes a prespecified primary outcome, a clearly defined limb-volume measure, patient-reported outcomes, prescribed and actual compression use, infection episodes, adverse events, and repeat interventions. A baseline clinical stage and the procedure performed are also recorded. ICG classification, when available, is reported separately from clinical stage.

The optional mechanistic module includes an assessment of anastomosis status, directed transport, and selected tissue characteristics. Quantitative flow is measured only when an appropriate method is available for the site and time point. Table S7 relates these elements to existing recommendations.

**Figure 2:**
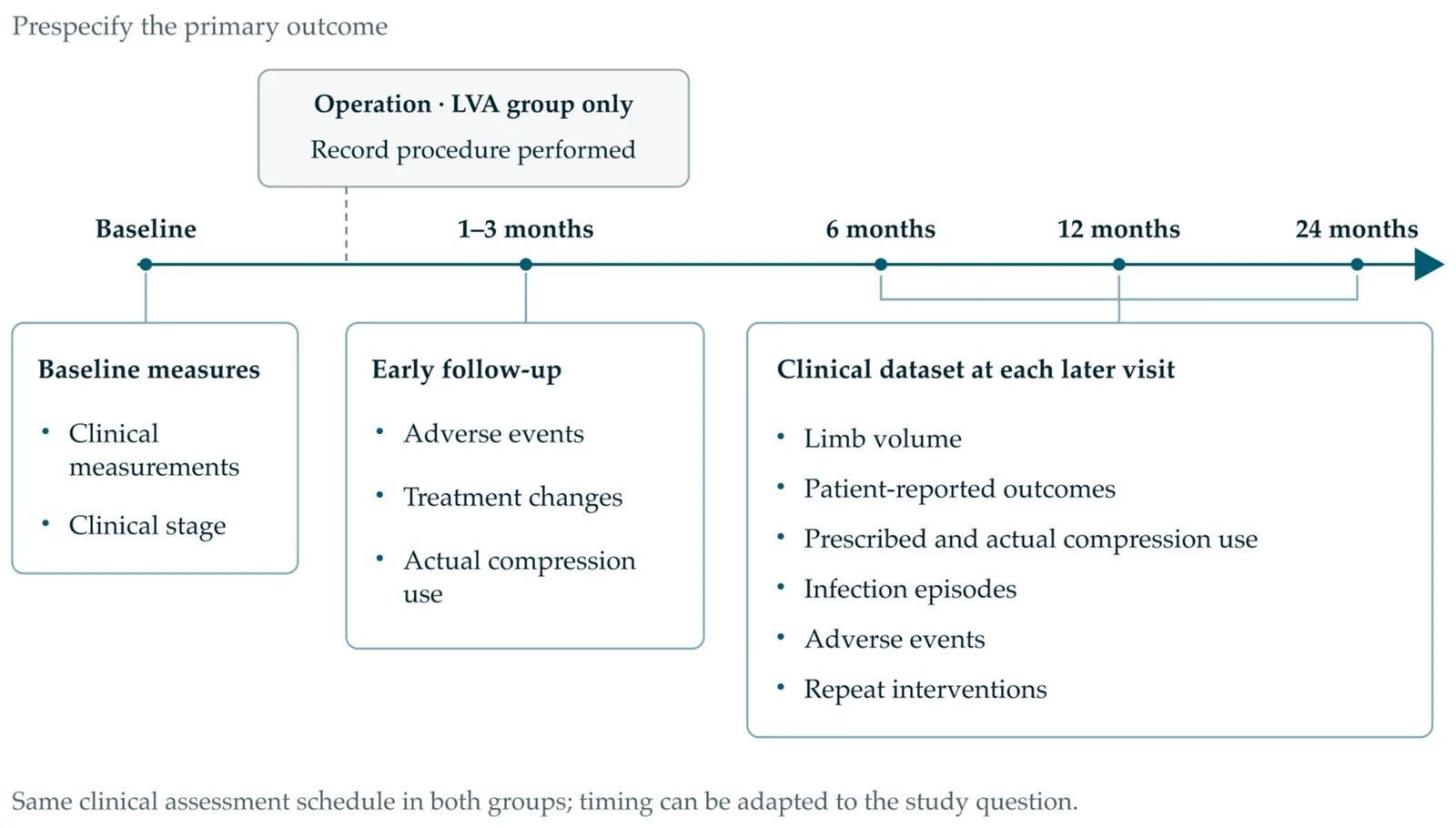
Proposed minimum clinical dataset and assessment schedule. This proposed research framework coordinates clinical measurements and compression documentation from baseline to 24 months. The primary outcome is prespecified as part of the study design. Comparative groups follow the same clinical assessment schedule; the operative record applies only to patients undergoing lymphaticovenular anastomosis (LVA). Timing can be adapted to the study question. The expanded framework, including optional mechanistic measurements, is presented in Figure S1.

A possible schedule includes baseline assessment, an operative record for patients undergoing LVA, early follow-ups at 1–3 months, and clinical assessments at 6, 12, and 24 months (Figure 2). Early follow-ups record adverse events, treatment changes, and actual compression use. Later assessments cover the clinical dataset described above. In comparative studies, both groups follow the same clinical assessment schedule. The timing can be adapted to the study question.

A possible mechanistic sequence adds early assessments of anastomosis status and transport, followed by tissue assessment at 6 months to explore associations with clinical outcomes at 12 and 24 months. Table S6 summarizes the proposed measurements, timing, and analytical roles.

### 8.3. Separate Analyses of Treatment Effect, Prognosis, and Mechanism

The proposed framework distinguishes three research questions: comparative treatment effect, mechanism, and prognosis after LVA (Table 3).

In randomized comparisons, the primary analysis includes patients according to their assigned treatment, regardless of technical completion or confirmed patency.

## 9. Limitations of the Review

This narrative review uses purposive selection of English-language publications and may be affected by selection and interpretation bias. Study selection and data extraction were not performed independently in duplicate, and no formal risk-of-bias or certainty assessment was conducted. The discussion and supplementary tables (Tables S1 and S2) concern the selected publications and should not be interpreted as a comprehensive assessment of the LVA literature.

Interpretation of the selected clinical studies is limited by the interim nature of some randomized analyses, differences in outcomes and comparators, missing data, and potential confounding in nonrandomized studies. Possible cohort overlap and reporting inconsistencies could not always be resolved from published reports. Reported improvements should be interpreted in this context.

The physiological and imaging findings discussed inform hypotheses about clinical response to LVA but do not establish its mechanism. The proposed framework has not been prospectively validated and is not a consensus standard or a patient selection rule.

## 10. Conclusions

The randomized findings reviewed here apply most directly to patients with unilateral BCRL and ICG-visible lymphatic vessels. Most trial participants had early-stage II changes, as defined by the authors. At six months, the physical domain of Lymph-ICF and reported compression use favored LVA added to conservative treatment. Between-group differences in the total score and volume measures were not convincing [5]. These findings suggest possible benefits in symptoms and treatment burden but do not establish sustained independence from compression. The two-year economic analysis does not replace detailed long-term clinical outcome data [14].

The proposed framework coordinates a minimum clinical dataset, including compression exposure, with optional mechanistic measurements. The review does not propose imaging or physiological measures as validated rules for selecting patients according to expected clinical benefits. Coordinated assessment of technical and clinical outcomes may help future studies establish when technical success after LVA translates into meaningful benefits for patients.

## Figures and Tables

**Table 1:** Principal comparative studies of therapeutic LVA for BCRL.

Study and Population	Intervention and Comparator	Follow-Up	Main Outcomes	Main Comparative Findings	Principal Limitations
Jonis et al., 2024 [5] Open-label RCT; unilateral BCRL with ICG-visible lymphatics. 100 randomized; 92 in the interim analysis.	LVA + conservative therapy versus conservative therapy. 46 per group at interim analysis.	6 months (interim analysis).	Primary: Lymph-ICF. Secondary: limb volume, UEL index, compression use.	Between-group total Lymph-ICF difference: −5.92 points (95% CI −14.88 to 3.04). Physical-domain difference: −13.77 points (*p* = 0.001). No convincing between-group difference in volume or UEL index. Compression: 10 complete and 10 partial discontinuations after LVA; none in controls.	Open-label interim analysis; multiple domains and outcomes. Most participants had author-defined stage IIa disease.
Shimbo et al., 2024 [6] Retrospective cohort; early-stage BCRL. 81 patients.	LVA + compression (*n* = 29) versus conservative treatment centered on compression (*n* = 52).	UEL index assessed at 6, 12, 18, and 24 months.	UEL index change; cellulitis incidence.	Reported group changes in UEL index at 24 months: −5.6% ± 6.0% versus +4.4% ± 10.7% (*p* < 0.001). Reported cellulitis incidence: 0% versus 15.4%.	Retrospective, single-center comparison with nonrandomized treatment selection.
Zeltzer et al., 2026 [7] Prospective nonrandomized cohort; BCRL. 92 patients across four groups.	LVA + CDT (*n* = 30) versus CDT alone (*n* = 34). Other arms: VLNT + CDT (*n* = 15); combined surgery + CDT (*n* = 13).	12 months.	Change in relative interlimb volume difference (RID).	Reported group mean RID changes: −6.21% ± 5.76% versus +3.23% ± 6.83% (*p* < 0.0001) for LVA + CDT versus CDT alone.	Nonrandomized treatment selection; controls had declined surgery (registry NCT06606145).
Seidenstuecker et al., 2024 [12] * Retrospective cohort; chronic BCRL. 105 patients.	LVA (*n* = 47) versus VLNT (*n* = 58).	24 months; 96/105 completed follow-up.	Circumference-based relative reduction; postoperative complications.	Group relative reduction rates: 2.14% ± 2.25% versus 2.64% ± 4.64% (*p* = 0.711). Postoperative complication events: 0 after LVA versus 11 after VLNT.	Nonrandomized procedure selection; larger baseline arm circumferences in the VLNT group.
Kappos et al., 2025 [13] * Retrospective cohort; chronic BCRL. 112 patients.	LVA (*n* = 42) versus VLNT (*n* = 70).	3–24 months; 107/112 completed 12-month follow-up.	Primary: circumference-based relative reduction over time.	Group-specific relative reduction at 24 months: LVA 1.84% (95% CI −0.79 to 4.48); VLNT 1.88% (95% CI 0.47 to 3.30). These CIs describe each group, not the between-group difference.	Nonrandomized procedure selection limits comparisons of the postoperative trajectories.

Results are presented as LVA versus comparator. Group-specific changes are distinguished from between-group effect estimates. UEL index, RID, and circumference-based reduction use different definitions and should not be treated as interchangeable measures. Kleeven et al. [14] reported a 24-month economic evaluation of the same randomized cohort as Jonis et al. [5]; it is not an independent clinical comparison. * Possible participant overlap between the Seidenstuecker and Kappos cohorts remains unconfirmed.

**Table 2:** Frameworks for the clinical role of LVA in BCRL and the basis for their recommendations.

Source	Document Type	Position on Therapeutic LVA	Stated Certainty of Evidence	Reported Risks and Limits on Access
International Society of Lymphology, 2023 [3]	International consensus document	Surgical methods are considered an adjunct to conservative treatment	Based on consensus opinion	Advanced stages usually require long-term physiotherapy and/or compression after surgery. Robust comparative evidence on long-term clinical benefit remains limited.
Lilja et al., 2024 [8]	Systematic review with an algorithm	LVA when functioning vessels are preserved, preferably in early stages	Low level of evidence. High risk of bias in the included studies	Greater uncertainty about the treatment effect of LVA than about that of liposuction or VLNT
Sacks et al., 2024 [77]	Expert working group consensus	Detailed requirements for candidate selection and preparation	Expert opinion. A formal consensus process is not described	Need for patient preparation, adherence, and realistic expectations
National Institute for Health and Care Excellence, 2025 [4]	Independent evidence assessment	Evidence is insufficient for firm conclusions about effectiveness, safety, and long-term outcomes. Potential benefit for selected patients is discussed through shared decision-making. LVA and VLNT are identified as areas requiring further comparative research	Evidence quality judged insufficient. Cost effectiveness remains uncertain	Limited number of centers and need for additional resources
Masià et al., 2026 [78]	Formal Delphi consensus	Emphasizes preoperative preparation, combined reconstructive and reductive methods, and multidisciplinary follow-up	Consensus threshold ≥70%, with a mean level of agreement of 79.6%. The treatment effect is not measured	Requires multidisciplinary infrastructure

**Table 3:** Research questions within the proposed assessment framework.

Question	What is Being Investigated	Role of Coordinated Assessment
Causal effect of the intervention	Does LVA improve clinical outcomes relative to a comparator treatment strategy?	Assess clinical outcomes at aligned follow-up times and document actual compression use in both groups.
Mechanism (mediation)	How might anastomotic function and tissue changes relate to clinical outcomes?	Use additional mechanistic measurements to examine the timing and sequence of functional, tissue, and clinical changes.
Prognosis of response among patients who undergo surgery	Which baseline or early postoperative characteristics help predict later clinical outcomes?	Relate these characteristics to subsequent outcomes among patients who undergo LVA.

Mechanistic measurements are additional to the minimum clinical dataset. Detailed methodological considerations and prognostic hypotheses are provided in Supplemental Methods S1.

## Data Availability

No new data were created or analyzed in this study. Data sharing is not applicable to this article.

## Supplementary Materials

The following supporting information can be downloaded at: https://www.mdpi.com/article/10.3390/jcm15207909/s1, Figure S1: Expanded research framework for therapeutic LVA in BCRL; Table S1: Comparative studies included in this narrative review that inform the assessment of clinical outcomes after therapeutic LVA; Table S2: Published evidence syntheses included in this narrative review, the types of data pooled, and the conclusions they support; Table S3: Technical and functional LVA statuses, definitions, and recommended denominators; Table S4: Possible sources of differences between study results; Table S5: Imaging and functional measurement methods, physical signals, research purposes, and limits of interpretation; Table S6: Variables in the sequential measurement framework, methods, timing, and analytical roles; Table S7: Existing clinical and research frameworks and the proposed addition; Supplemental Methods S1: Treatment effect, prognosis, and investigation of mechanism. References [3,5,6,7,8,9,10,11,12,13,14,15,24,29,31,32,33,34,35,36,37,38,39,46,57,58,59,62,63,67,68,71,74,75,76,77,78,86,87,88,89] are cited in the Supplementary Materials.

## Abbreviations

The following abbreviations are used in this manuscript:

AGReMA	A Guideline for Reporting Mediation Analyses
BCRL	Breast cancer-related lymphedema
BIS	Bioimpedance spectroscopy
BSO	Backflow, slack, and outlet classification
CDT	Complex decongestive therapy
CE-MRL	Contrast-enhanced magnetic resonance lymphangiography
CEUS	Contrast-enhanced ultrasound
GRADE	Grading of Recommendations Assessment, Development and Evaluation
CI	Confidence interval
SMD	Standardized mean difference
AUC	Area under the curve
ICG	Indocyanine green
ISL	International Society of Lymphology
L-Dex	Lymphedema index
LVA	Lymphaticovenular anastomosis
LVB	Lymphaticovenous bypass
Lymph-ICF	Lymphedema Functioning, Disability and Health Questionnaire
Lymph-ICF-UL	Lymphedema Functioning, Disability and Health Questionnaire for Upper Limb Lymphedema
MRL	Magnetic resonance lymphangiography
NECST	Normal, ectasis, contraction, and sclerosis type classification
NICE	National Institute for Health and Care Excellence
NIRF	Near-infrared fluorescence
QALY	Quality-adjusted life-year
RID	Relative interlimb volume difference
ROC	Receiver operating characteristic
SWE	Shear wave elastography
UEL index	Upper extremity lymphedema index
UHFUS	Ultra-high-frequency ultrasound
VLNT	Vascularized lymph node transfer
