# Supplementary material for: Therapeutic Lymphaticovenular Anastomosis for Breast Cancer-Related Lymphedema: A Critical Narrative Review of Comparative Evidence
Source: J Clin Med. 2026 Oct 13;15(20):7909. doi: 10.3390/jcm15207909 (PMC13705796; doi:10.3390/jcm15207909)
Supplement: Supplementary file 1 [file jcm-15-07909-s001.zip › jcm-4585992-supplementary.pdf]

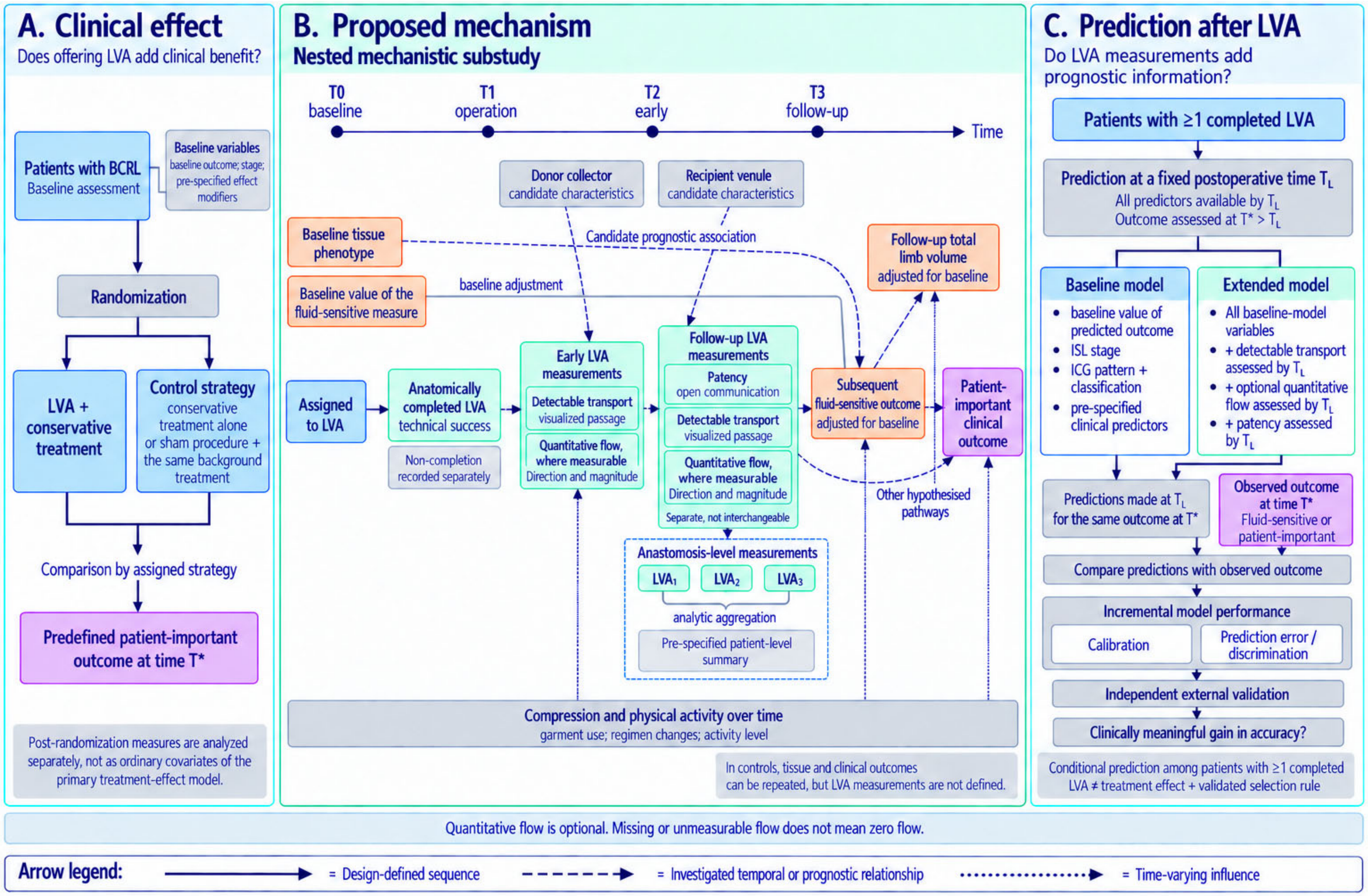

Figure S1. Expanded research framework for therapeutic LVA in BCRL. The diagram complements the minimum clinical dataset and assessment schedule in Figure 2. (A) Comparative treatment effects assessed by assigned strategy. (B) An optional mechanistic substudy relating operative, early and follow-up measurements to tissue characteristics and clinical outcomes. (C) Postoperative prognosis among patients with at least one completed LVA, using predictors available by a prespecified time T_L to predict an outcome assessed later, at $T^* > T_L$. Quantitative flow measurement is optional; missing or unmeasurable flow does not indicate zero flow. Arrows represent design sequences or hypothesized relationships, not proof of causality. Postoperative prognostic associations do not establish comparative treatment effects or validated rules for selecting patients for surgery.

Table S1. Comparative studies included in this narrative review that inform the assessment of clinical outcomes after therapeutic LVA.

Study (publication)	Design, groups, and follow-up	Population and directness of evidence for BCRL	Clinical question addressed by the comparison	Observed estimate (95% CI where reported)	Design and reporting features relevant to interpretation	Related publications or cohort overlap
Jonis et al., 2024 [1]	Open-label RCT. 100 patients randomized, with 6-month data available for 92 (46/46). LVA plus conservative treatment versus conservative treatment. Follow-up of 6 months, with the primary outcome prespecified for 12 months.	Direct evidence. Upper-extremity BCRL, ISL stage I or the authors' subcategory "IIa," and Narushima ICG stages II–III.	Addition of LVA to conservative treatment. Quality of life and volume.	The between-group difference in the total Lymph-ICF score was −5.92 points (95% CI −14.88 to 3.04, p = 0.192), and the difference in the physical domain was −13.77 points (p = 0.001, see the note on the CI). For the volume measure obtained by water displacement, the authors reported no statistically significant between-group difference (p = 0.737). The difference in the UEL-index was 2.43 (p = 0.458). In the LVA group, 10 patients stopped using compression garments completely and 10 reduced their use. No such changes were recorded in the control group.	Open-label RCT without sham surgery. Awareness of treatment assignment may have influenced self-reported outcomes. The 6-month analysis was interim. Of 100 patients, 92 had reached this time point, while the other eight had not yet completed follow-up. One dropout was reported. No strategy for multiple comparisons was described, and no CI was provided for the volume outcome. There were discrepancies in ICG stages and denominators for the compression outcome. The LVA technique was partly described, including end-to-end anastomoses, 11-0 sutures, 1–5 anastomoses, and intraoperative confirmation of patency. This publication did not report results of a systematic assessment of late patency.	Kleeven et al., 2026 used the same sample for a 24-month economic analysis. The authors stated that final clinical results would be reported in a separate publication.

Study (publication)	Design, groups, and follow-up	Population and directness of evidence for BCRL	Clinical question addressed by the comparison	Observed estimate (95% CI where reported)	Design and reporting features relevant to interpretation	Related publications or cohort overlap
Kleeven et al., 2026 [2]	Prespecified secondary economic analysis of the same RCT. 100 patients (50/50). Follow-up of 24 months. Intention-to-treat analysis with multiple imputation.	Direct evidence. The same patients.	Costs and QALYs with the addition of LVA	QALY difference of 0.045 (−0.021 to 0.112). Cost difference of €78 (−6044 to 6200). Probabilities of cost-effectiveness were 0.60 and 0.71 from the societal perspective and 0.08 and 0.42 from the healthcare system perspective at thresholds of €20,000 and €50,000.	A secondary economic analysis of the same randomized cohort, not an independent study. The analysis followed the intention-to-treat principle and used multiple imputation. QALYs and costs do not replace disease-specific clinical outcomes. At baseline, 47/50 patients in the LVA group and 42/50 in the control group used compression garments. Aggregate use of compression garments and bandages was similar between groups in the economic analysis. This measure does not establish that actual hours of wear or sustained independence from compression were similar. The authors stated that detailed disease-specific clinical results would be reported in a separate publication.	Jonis et al., 2024 used the same cohort.

Study (publication)	Design, groups, and follow-up	Population and directness of evidence for BCRL	Clinical question addressed by the comparison	Observed estimate (95% CI where reported)	Design and reporting features relevant to interpretation	Related publications or cohort overlap
Mihara et al., 2024 [3]	Multicenter RCT. 336 participants randomized. LVA plus conservative therapy versus conservative therapy. Follow-up of 6 months.	Indirect evidence. Secondary lower-extremity lymphedema.	Addition of LVA. Frequency of soft tissue infections.	The between-group difference in the change in the number of cellulitis episodes over 6 months was −0.35 (95% CI −0.62 to −0.09, $p = 0.010$) in the authors' full-analysis set of 225 of the 336 randomized participants and −0.60 (−0.94 to −0.27, $p = 0.001$) in the per-protocol set ($n = 156$). No statistically significant differences in circumference or pain were found. Secondary outcomes were available for 170 participants in the full-analysis set. Palpable stiffness decreased more after LVA in some thigh regions. This measure is not a direct assessment of fibrosis or tissue water.	A multicenter RCT, but only 225 of the 336 randomized participants were included in the main cellulitis analysis and 156 in the per-protocol analysis. Concomitant treatment differed after randomization. CDT was intensified in 8 patients in the LVA group and 26 in the control group, and 10 patients undergoing LVA also had genital lymphatic vesicles removed. The study population had lower-extremity lymphedema, so the evidence is indirect for BCRL.	–
Shimbo et al., 2024 [4]	Retrospective comparative study. Of 101 patients, 81 were included in the analysis. LVA plus compression, $n = 29$. Conservative treatment, $n = 52$. UEL-index assessments at 6, 12, 18, and 24 months. Mean follow-up was 32.9 ± 19.0 months in the LVA group and 51.2 ± 35.6 months in the conservative group.	Direct evidence. Early-stage BCRL.	Treatment strategy selected in clinical practice. Relative change in the UEL-index.	At 6 months, −6.6 ± 7.3% versus +0.9 ± 7.5%. At 12 months, −7.3 ± 6.2% versus +2.9 ± 8.6%. At 18 months, −7.6 ± 8.0% versus +3.9 ± 9.2%. At 24 months, −5.6 ± 6.0% versus +4.4 ± 10.7%. All time points had $p < 0.001$. Cellulitis after LVA or the first conservative visit was reported in 0/29 versus 8/52 patients ($p = 0.026$).	A single-center retrospective cohort. Of 101 patients, 81 were included in the main comparison. Treatment was selected clinically, with no adjustment for baseline differences described. Actual compression exposure was not quantified. Cellulitis findings are limited by few events and unequal follow-up between groups. The observed differences support an association with the selected strategy rather than a causal effect of LVA.	–

Study (publication)	Design, groups, and follow-up	Population and directness of evidence for BCRL	Clinical question addressed by the comparison	Observed estimate (95% CI where reported)	Design and reporting features relevant to interpretation	Related publications or cohort overlap
Seidenstuecker et al., 2024 [5]	Retrospective comparative study. 105 patients, with 47 undergoing LVA and 58 undergoing VLNT. Of 105 patients, 96 (91.4%) had at least 24 months of follow-up.	Direct evidence. Chronic BCRL.	LVA versus VLNT, without a nonsurgical control group. Relative reduction based on circumference measurements and complications.	Relative reduction, as expressed in the original publication, was 2.14 ± 2.25 with LVA versus 2.64 ± 4.64 with VLNT ($p = 0.711$). Postoperative complications according to Clavien–Dindo were 0 with LVA versus 11 (19.0%) with VLNT.	A retrospective, nonrandomized comparison of LVA and VLNT without a nonsurgical control group. The groups differed in disease severity at baseline. Follow-up of at least 24 months was completed by 96/105 patients. The LVA technique was described, with a mean of 2.4 ± 0.7 anastomoses, predominantly side-to-end. Outcomes were assessed mainly using circumference measurements and Clavien–Dindo, without a validated PROM.	Kappos et al., 2025 likely used a related institutional database. The extent of participant overlap has not been established, so the independence of the cohorts remains uncertain.
Kappos et al., 2025 [6]	Retrospective cohort. 112 patients, with 42 undergoing LVA and 70 undergoing VLNT. One-year follow-up was completed by 107 (95.5%). Follow-up of 3–24 months.	Direct evidence. Chronic BCRL.	LVA versus VLNT, without a nonsurgical control group. Calculated reduction based on circumference measurements over time.	Different time courses. LVA showed an early peak within the first 3 months followed by a decline. VLNT showed two peaks, at 3 and 18 months. Point estimates with CIs for the between-group comparison were not reported.	A retrospective analysis of a prospectively maintained database. Selection of LVA or VLNT was not randomized. Of 112 patients, 107 completed at least 12 months of follow-up, but the number of participants at 18–24 months was not reported. Baseline circumference was somewhat greater in the VLNT group. Follow-up ICG imaging was not performed systematically, and patient-important outcomes were not assessed.	Seidenstuecker et al., 2024, as noted above. The institutional databases may be related, but the extent of overlap has not been established.

Study (publication)	Design, groups, and follow-up	Population and directness of evidence for BCRL	Clinical question addressed by the comparison	Observed estimate (95% CI where reported)	Design and reporting features relevant to interpretation	Related publications or cohort overlap
Zeltzer et al., 2026 [7]	Prospective, nonrandomized controlled study. 92 patients, with 30 undergoing LVA, 15 undergoing VLNT, 13 undergoing the combination, and 34 receiving conservative treatment. Follow-up of 12 months.	Direct evidence. BCRL.	Patients who agreed to surgery versus those who declined. Change in RID.	LVA, −6.21% ± 5.76%. VLNT, −9.61% ± 12.03%. Combination, −7.00% ± 6.56%. Control, +3.23% ± 6.83%. Each surgical group versus control had $p < 0.0001$.	Prospective outcome collection with nonrandomized treatment assignment. The control group consisted of patients who declined surgery. Registration occurred after recruitment began. The registry lists 179 participants, whereas the publication analyzes 92, and the reason for this discrepancy is unclear. The actual intensity of concomitant CDT was described only to a limited extent.	–

Note. In Table 3 of the publication by Jonis et al., the upper bound of the 95% CI for the physical domain is printed as +5.69, with an estimate of −13.77 and $p = 0.001$. These values are internally inconsistent. A presumed correction of the sign is not reproduced here as a confirmed value from the authors. For the between-group volume measure of −2.82, the unit and calculation method are not presented clearly enough. The Jonis report also gives inconsistent ICG stage III counts. The text reports 85 of 92 patients, whereas Table 1 reports 75 of 91 patients with available staging data. These published counts do not permit an unambiguous reconstruction of the stage distribution.

Table S2. Published evidence syntheses included in this narrative review, the types of data pooled, and the conclusions they support.

Evidence synthesis	Included evidence	Clinical question and population	Main findings	Type of comparison pooled	Limitations on conclusions about the causal effect of LVA
Rosian & Stanak, 2019 [8]	5 prospective studies with 217 patients. One nonrandomized controlled study and four single-arm series.	Effectiveness and safety of therapeutic LVA for primary and secondary lymphedema. The population extends beyond BCRL.	The certainty of evidence for effectiveness and safety was rated very low. No reliable conclusion about the comparative effectiveness of LVA can be drawn.	Predominantly before-and-after changes. One nonrandomized comparison of LVA with vascularized lymph node transfer.	All included studies had a moderate or high risk of bias. Most data were uncontrolled, and direct comparisons were limited. The results do not provide a BCRL-specific estimate of the treatment effect.
Chang et al., 2021 [9]	Systematic review and meta-analysis of controlled studies and uncontrolled case series of surgical treatment and prevention of lymphedema, with consensus recommendations.	Surgical treatment and prevention of upper- and lower-extremity lymphedema. LVA, VLNT, and reductive procedures. The population extends beyond BCRL.	The evidence supports a possible reduction in lymphedema severity after LVA, graded 1C by the authors. No consensus was reached on the superiority of LVA or VLNT, graded 2C.	Between-group comparisons and within-group pre/post changes, depending on the procedure and outcome. Consensus interpretation of the findings.	The authors rated the evidence for the pooled reduction in circumference after LVA combined with compression therapy as very low. Populations and procedures varied. A lack of consensus on the superiority of LVA or VLNT does not establish equivalence. Prevention findings cannot be applied directly to the treatment of established BCRL.
Meuli et al., 2023 [10]	150 studies with 6496 patients. LVA and VLNT.	Treatment. Mixed anatomical sites and etiologies.	Change in excess circumference of −35.6% (95% CI −40.3 to −30.8) across 29 studies and 1002 patients.	Predominantly before-and-after changes.	The estimate applies to microsurgery overall, not specifically to LVA.
Lilja et al., 2024 [11]	73 BCRL-specific studies with 2373 patients. Formal risk-of-bias assessment.	Treatment. BCRL only.	An algorithm for choosing among liposuction, LVA, and VLNT. The effect of LVA is the most uncertain.	Mixed, predominantly observational.	The included studies had a high risk of bias. The authors proposed the algorithm based on a qualitative synthesis of the available evidence.
Zapata-Ospina et al., 2024 [12]	5 observational studies. Included publications encompass the large mixed series by Campisi.	The stated question concerns LVA combined with CDT for BCRL. The actual evidence base also includes lower-extremity lymphedema.	Changes were in a favorable direction. In the full text, the authors rated the certainty of evidence on lymphedema reduction and quality of life as very low.	Four before-and-after studies and one observational cohort.	Most data were uncontrolled, and part of the population provided indirect evidence. Assessment used NHLBI tools. The independent comparative effect of LVA has not been established.

Evidence synthesis	Included evidence	Clinical question and population	Main findings	Type of comparison pooled	Limitations on conclusions about the causal effect of LVA
Hahn BA et al., 2025 [13]	52 studies with 1920 limbs.	Treatment. Upper and lower extremities with different etiologies.	Mean relative reduction in excess circumference or volume after LVA was 29.44% (95% CI 15.58 to 43.29) for the upper extremity and 31.87% (95% CI 18.60 to 45.14) for the lower extremity. The reduction after VLNT for the upper extremity was 41.66% (95% CI 34.13 to 49.20).	Pooling of within-group postoperative changes. Comparison of study subgroups by procedure.	The reported estimates are within-group changes and do not establish the comparative superiority of the procedures. Circumference and volume changes are pooled across highly heterogeneous studies.
Zurfluh et al., 2025 [14]	74 studies with 4692 patients. Various lymphatic reconstructive procedures.	Treatment. Mixed anatomical sites and etiologies.	Improvements beyond volume, including quality of life and infection outcomes.	Mixed.	Combining different procedures, etiologies, and anatomical sites.
Jungbauer et al., 2025 [15]	23 studies with 648 limbs. Follow-up of at least 24 months.	Treatment. Secondary lymphedema overall, not only BCRL.	The pooled mean change in annual cellulitis episodes after LVA was −1.13 (95% CI −1.70 to −0.57) for the upper extremity.	Predominantly within-group comparisons.	Secondary lymphedema overall, not only BCRL. The comparative effect cannot be identified.
Brown et al., 2026 [16]	8 prospective studies from 2009–2023 with 431 patients.	Treatment. Secondary upper- and lower-extremity lymphedema, not only BCRL.	Limb volume decreased by 14.26% at 1 year. Compression was reduced or discontinued in 46.3%. The mean number of anastomoses per patient was 3.4 ± 1.0.	Prospective single-arm studies.	"Prospective" does not mean "randomized." The population extends beyond BCRL. Pooled changes are not equivalent to a comparative treatment effect.
Kumar et al., 2026 [17]	13 studies. Therapeutic LVA in 9 studies and prophylactic LVA in 4.	Treatment and prevention were analyzed separately. BCRL.	Quality of life SMD of 0.86 (95% CI 0.62 to 1.10). Physical domain, 1.54. Mental domain, 1.03.	Within-group before-and-after changes, including data from the surgical arm of a randomized trial.	Describes postoperative change within groups rather than an effect relative to a control group.
Zhang and Lodi, 2026 [18]	Network meta-analysis of immediate and delayed lymphatic reconstruction.	BCRL. Therapeutic and prophylactic strategies were considered in the same publication.	Therapeutic comparisons did not establish convincing superiority of LVA or VLNT. Prevention analyses addressed the risk of developing BCRL.	Direct and indirect network comparisons. Interpretation depends on the structure of the specific network.	Dependent on transitivity and indirect comparisons. Absence of a difference does not establish equivalence. Prevention and treatment questions are not interchangeable.

Note. The evidence certainty categories and the designations 1C and 2C reproduce the judgments of the authors of the respective evidence syntheses. No formal reassessment was conducted in this narrative review.

Table S3. Technical and functional LVA statuses, definitions, and recommended denominators.

Event or status	Definition and recommended denominator
Mapping	Record separately when a lymphatic vessel cannot be located, when no suitable collector–venule pair is found, and when planned mapping is not performed. The denominator is the number of sites and/or patients examined, with the number of planned assessments also reported.
Technical completion	The anastomosis is anatomically complete according to a prespecified operative criterion. Do not combine this status with directional transport or late patency. The denominators are attempted anastomoses and patients in whom surgery was started.
Confirmed directional transport	The positive criterion for transport direction is met using the selected method under specified conditions. The method and time point must be reported. The denominator is completed anastomoses or, for patient-level analysis, patients whose transport was assessed.
Confirmed patency	Persistence of an open connection is confirmed by a prespecified method at a specific postoperative time point. Report n/N separately for anastomoses and for patients with at least 1 patent anastomosis.
Confirmed occlusion	The available evidence meets the prespecified criterion for occlusion. Failure to visualize the anastomosis alone is insufficient without such a criterion.
Indeterminate assessment	The examination was performed, but its result does not allow classification of transport, patency, or occlusion. Retain this as a separate category.
Not assessed	The planned examination was not performed. Report the reason, if known. Do not code this as absent flow or occlusion.
Lost to follow-up	Subsequent status is unknown because the participant cannot be reached. Distinguish this from a completed examination with an indeterminate result and from refusal of an individual procedure.

Note. At each time point, report separately the number of anastomoses and patients due for assessment, the number actually examined, and the distribution of results. For each proportion n/N, explicitly state whether the denominator includes everyone due for assessment or only those examined. Do not combine indeterminate results, examinations not performed, or losses to follow-up with confirmed occlusion.

Abbreviations. BCRL, breast cancer-related lymphedema. LVA, lymphaticovenular anastomosis. VLNT, vascularized lymph node transfer. CDT, complex decongestive therapy. PROM, patient-reported outcome measure. HRQoL, health-related quality of life. QALY, quality-adjusted life-year. ICG, indocyanine green. NIRF, near-infrared fluorescence. CEUS, contrast-enhanced ultrasound. UHFUS, ultra-high-frequency ultrasound. MRL, magnetic resonance lymphangiography. SWE, shear wave elastography. BIS, bioimpedance spectroscopy. ISL, International Society of Lymphology. CI, confidence interval. Instrument names and author-defined indices retain their original spelling.

Table S4. Possible sources of differences between study results.

Possible source of differences	Findings in the publications included in this review	What remains uncertain	Status of the explanation	What should be measured or reported
Volume metrics and breadth of the construct	The included comparative publications used different volume metrics and follow-up periods ranging from approximately 3 to 24 months.	The proportion of differences between studies attributable to the choice of metric and its sensitivity to change.	A documented difference. The influence of tissue composition on the volume response remains a hypothesis.	A prespecified volume formula and standardized measurement conditions. A separate fluid-sensitive measure in mechanistic studies.
Differences in research questions	Comparisons of LVA with conservative treatment, LVA with VLNT, and mapping methods address different research questions. Randomized and nonrandomized studies can seek to estimate the same comparative treatment effect while differing in their protection against confounding. A single-arm before-and-after change does not, by itself, estimate a between-group contrast.	The extent to which estimates differ numerically across designs and control strategies.	A documented difference.	Clearly specify the population, strategies being compared, outcome, and assessment time point. Do not conflate between-group treatment effects with within-group changes.
Assessment time points	Assessment time points differ. One nonrandomized cohort described a nonmonotonic time course after LVA and VLNT.	Whether later differences reflect the treatment effect, natural changes over time, or changes in concomitant therapy.	A documented difference and a plausible explanation.	A prespecified assessment schedule for clinical and functional measures.
Clinical selection and confounding by indication	In Zeltzer, the control group was defined by refusal of surgery. In Shimbo and Kappos, the strategy was selected clinically rather than through randomization.	The direction and magnitude of selection-related bias.	A documented design feature.	Randomization or detailed recording of factors influencing treatment selection and the outcome, justified adjustment for confounding, and a prespecified sensitivity analysis.
Compression therapy	Protocols and reporting differ. In the publications included in this review, detailed longitudinal data on actual compression exposure, including hours of wear, compression class, and regimen changes, were incomplete.	The extent to which changes in compression are associated with volume and patient-important outcomes after LVA.	A plausible explanation. Its quantitative contribution has not been established.	Longitudinal recording of compression type, class, hours of wear, and reasons for changing and resuming compression.

Possible source of differences	Findings in the publications included in this review	What remains uncertain	Status of the explanation	What should be measured or reported
Technical features and extent of the procedure	Technical parameters are reported inconsistently. Jonis describes end-to-end LVA with 1–5 anastomoses. Seidenstuecker reports predominantly side-to-end anastomoses and the mean number performed. In Gong, the number of anastomoses differed between groups. [19]	Whether the configuration, caliber, number, and functional adequacy of anastomoses are associated with subsequent outcomes.	Inconsistent reporting is documented. The clinical significance remains a hypothesis.	Record planned and completed anastomoses, configuration, and caliber at the anastomosis level, with a prespecified approach to aggregation at the patient level.
Tissue phenotype	Individual derived measures have been reported, such as tissue "stiffness" in lower-extremity lymphedema and segmental water content. However, in the included comparative publications, we found no combined assessment that quantified fluid, adipose tissue, and matrix separately.	Whether baseline tissue phenotype modifies the comparative treatment effect of LVA.	A biologically plausible but untested hypothesis.	One prespecified fluid-sensitive measure. Standardized assessment of adipose and mechanical components when these are the subject of a separate research question.
Range of stages and interaction analyses	The BCRL-specific RCT was nearly homogeneous in the early Stage II subcategory defined by the authors. Interaction analyses were not the study's primary focus.	Whether any baseline characteristics modify the comparative treatment effect of LVA.	The limited range of baseline characteristics is documented. Treatment effect modification has not been established.	Prespecified analyses of treatment × baseline characteristic interactions, with a sample size calculated for the interaction.
Reporting and possible publication bias	Some sources contain internal discrepancies, retrospective registration, or incompletely described denominators.	The extent of selective publication of negative results and technical failures.	Specific reporting problems are documented. Publication bias was not formally assessed.	Prospective protocol registration, complete denominators, and publication of technical failures and negative results.

Table S5. Imaging and functional measurement methods, physical signals, research purposes, and limits of interpretation.

Method	Physical measurement	Research purpose	What has been established	What remains unestablished or the key limitation
ICG lymphography / NIRF	Superficial lymphatic pathways, dermal backflow, and fluorescence dynamics	Superficial mapping, characterization of dermal backflow, and technical planning	The method is clinically applicable for imaging superficial lymphatic anatomy and dermal backflow patterns. [20]	It is not a calibrated measure of overall transport reserve, tissue composition, or individual benefit from LVA. It is limited by imaging depth and differences among staging systems.
Conventional / high-frequency ultrasound (~18–24 MHz)	The lumen and wall of an accessible vessel, and sometimes observable contractility	Preoperative mapping of the lymphatic collector and recipient venule	Technical feasibility has been demonstrated, including detection of lymphatic vessels at 92.6% of examined sites in a small BCRL series. [21]	Equivalence or added value relative to ICG and the ability to predict clinical outcomes have not been established. The method is operator dependent.
UHFUS, with frequency reported for the specific transducer	Morphology of superficial submillimeter lymphatic vessels and venules without contrast	Preoperative selection of a lymphatic vessel–venule pair	In the series by Kim et al. (2025), 32/33 preselected pairs were confirmed intraoperatively. Tang et al. (2026) examined combined CEUS and ultrasound planning using a 24 MHz transducer, termed UHFUS by those authors. [22,23]	A high confirmation rate for positive findings does not establish sensitivity for all suitable vessels. Comparative clinical benefit has not been established, and imaging depth and results depend on the operator.
CEUS	Movement of intradermally injected microbubbles through lymphatic pathways	Mapping at greater depth and selection of the LVA site	Technical applicability has been demonstrated. In the chronological cohort by Gong et al. (2025), which included 52 patients with BCRL, ICG+CEUS (n = 30) was compared with ICG (n = 22). Anastomoses were completed in 154/185 and 71/112 explored incisions, respectively. This is a technical outcome, not a measure of late patency. Combined CEUS and ultrasound mapping with a 24 MHz transducer was also examined in the pilot series by Tang et al. [19,23]	The method's independent contribution to patient-important outcomes has not been established. Available comparative series are small and/or sequential in time, and the procedure requires contrast. Analyses of incisions must account for their dependence within patients.
Quantitative flowmetry using transit time	Local volumetric flow measured perioperatively before and immediately after LVA creation (mL/min)	Quantitative assessment of local transport as a candidate mechanistic measure	Perioperative quantitative measurement was technically feasible in a small series. [24]	It does not measure the additional total lymphatic clearance of the entire limb. Longitudinal reproducibility and associations with clinical outcomes have not been established. The series by Miseré found no statistically significant increase in flow immediately after LVA.

Method	Physical measurement	Research purpose	What has been established	What remains unestablished or the key limitation
Lymphoscintigraphy	Regional radiopharmaceutical transport with limited spatial detail	Assessment of regional transport function and postoperative changes	The method is used to assess lymphatic transport and diagnose lymphedema. [21]	It does not measure local flow through each LVA and has not been validated as an individual predictor of the comparative benefit of LVA.
Noncontrast MRL / T2-weighted MRI	Fluid-sensitive signal and tissue morphology	Assessment of edema distribution and tissue signal	It allows characterization of fluid-sensitive and morphological changes without interstitial contrast.	It does not directly visualize a functioning lymphatic collector or quantitatively measure tissue water. Its prognostic value for LVA has not been established.
Contrast-enhanced MRL	Anatomy of lymphatic pathways and interstitial contrast kinetics	Anatomical mapping at greater depth and LVA planning	Its technical value for localization is supported by case series. In Zhu et al. (2025), 150 of 151 vessels visualized by CE-MRL were identified and anastomosed intraoperatively. [25]	The data are predominantly retrospective and include mixed anatomical sites. Prediction of individual patient-important benefit has not been established. Protocols are heterogeneous, and interstitial gadolinium is used.
SWE and B-mode ultrasound	Mechanical tissue response and the thickness of individual layers	Characterization of mechanical and structural tissue properties	A local association between SWE and stage has been demonstrated in BCRL, with an AUC of 0.80 for forearm skin in He et al. (2026). [26]	SWE does not specifically distinguish water, pressure, and fibrosis. It depends on the region, tissue layer, and technique and has not been validated for predicting response to LVA.
BIS and derived indices	An indirect electrical measure sensitive to extracellular fluid distribution	Fluid-sensitive outcome or baseline descriptor	Randomized evidence for BIS is available in the context of surveillance and early compression intervention for BCRL. [27]	It does not directly measure absolute water content and has not been validated as a mediator, treatment effect modifier, or selection rule for LVA.
MRI–CEUS fusion	Integration of MR mapping of lymphatic pathways with contrast-enhanced ultrasound	Preoperative mapping and technical planning	Technical feasibility has been demonstrated retrospectively by Li Y et al. (2024). [28]	The sample includes mixed anatomical sites and etiologies. Added clinical value and prediction of response have not been established.
Photoacoustic imaging	An optoacoustic signal from contrast in the anastomotic region	Postoperative imaging of transport or anastomotic status	It is being investigated as a method for postoperative assessment of the LVA region by Suzuki et al. (2025). [29]	It does not automatically establish volumetric flow rate or sustained transport. Sensitivity is limited when flow is intermittent or contrast concentrations are low. Its validity as a clinical surrogate has not been established.

Table S6. Variables in the sequential measurement framework, methods, timing, and analytical roles.

Variable	Method, units, and timing	Measurement requirements	Analytical role	Current status
Candidate characteristics of the donor lymphatic collector	ICG pattern with the staging system specified. Ultrasound assessment of the wall and lumen. Observed contractility. Intraoperative macroscopic morphology according to NECST. Measured preoperatively and/or intraoperatively, depending on the method.	Do not combine different proxies into a single measure of "transport capacity" without validation. Standardize the protocol and assess reproducibility.	Technical suitability and candidate prognostic features among surgical patients. Modification of the comparative treatment effect is assessed using baseline characteristics measured in the same way in the groups being compared. Intraoperative NECST alone does not support such an analysis.	ICG is applicable for mapping, and ultrasound is technically feasible. NECST is a macroscopic classification with histological correlates developed outside BCRL. No validated rule for predicting response is available. [30]
Local hemodynamics of the recipient venule	Direction of transport or reflux, venule caliber, and valves near the anastomosis. Local pressure or pressure gradient, where feasible. Measured intraoperatively with the limb in a standardized position.	Record limb position, observation duration, and anesthetic conditions. Do not equate the visual BSO phenotype with a direct pressure measurement.	A candidate predictor of technical completion and early transport. Its prognostic role among surgical patients requires validation.	BSO standardizes visual description. It has not been validated as a pressure measure or undergone external validation as a predictor of clinical outcomes. [31]
Proximal venous outflow	Ultrasound or CT venography as clinically indicated. Presence and severity of proximal venous disease.	Analyze separately from local venular hemodynamics. Do not infer recipient venule pressure from an anatomical stenosis.	A candidate baseline prognostic factor. A potential confounder in nonrandomized comparisons if associated with both treatment selection and the clinical outcome.	Measurement is technically feasible. An association between correction of proximal disease and LVA patency or clinical benefit has not been established.
Actual delivery of the procedure	Number of planned, attempted, and completed LVAs. Configuration and vessel caliber for each anastomosis. Measured intraoperatively.	Define technical completion separately from directional transport and late patency. Account for anastomoses being nested within patients.	A postrandomization characteristic of intervention delivery. A technical outcome.	Can be measured using standard methods, but technical parameters and denominators are reported inconsistently in the publications included in this review.
Directional transport and subsequent patency	Qualitative or imaging confirmation of transport direction. Quantitative local flowmetry if a valid method is available. Patency is assessed separately at prespecified time points.	For each method, define criteria for positive, negative, and indeterminate results. Assess reproducibility and specify the level of analysis.	Candidate intermediate mechanistic measures. They do not replace a patient-important outcome.	Local quantitative measurement is possible perioperatively, but no standardized longitudinal method or validation as a clinical surrogate is yet available. [24]

Variable	Method, units, and timing	Measurement requirements	Analytical role	Current status
Compression and external influences on lymph flow	Compression type and class, hours of wear, and reasons for regimen changes. Physical activity and limb position during functional measurements. Recorded longitudinally.	Record the baseline regimen, postoperative prescription, actual compression exposure, reduction or discontinuation, and resumption separately.	A baseline factor, a time-varying concomitant intervention, and/or a patient-important outcome, depending on the research question.	In the publications included in this review, compression prescriptions are described inconsistently, and detailed data on actual compression exposure over time are incomplete.
Candidate fluid-sensitive measure	One prespecified method, such as BIS/L-Dex, segmental assessment of extracellular water, pitting, or a specified T2 MRI measure. Assessed before surgery and at a prespecified follow-up time point.	Do not combine different modalities into a single "water fraction." Standardize time of day, position, and the interval after compression removal.	A candidate baseline prognostic factor and an outcome in mechanistic studies. Treatment effect modification requires assessment of an interaction in a comparative analysis.	The methods measure different constructs. BIS has an evidence base for BCRL surveillance but has not been validated as a measure of the reversible fluid fraction or a mediator of the LVA treatment effect. [27]
Adipose tissue and subcutaneous layer thickness	Quantitative MRI assessment of fat fraction and/or standardized measurement of subcutaneous layer thickness.	Fixed anatomical sites and a uniform protocol. Do not treat layer thickness as equivalent to fat fraction.	A candidate baseline prognostic factor or characteristic of tissue phenotype.	Technically feasible measures. Their added prognostic value for LVA has not been established.
Matrix and mechanical tissue properties	SWE in prespecified tissue layers and regions. Histology only in studies specifically designed for that purpose.	Record the region and tissue layer separately. Do not interpret SWE as a direct measure of fibrosis content.	A candidate baseline prognostic factor and a tissue outcome in research.	SWE is a composite proxy for mechanical properties. Clinical prognostic validation for LVA is lacking.
Patient-important clinical outcome	Prespecify the primary outcome. Obtain baseline clinical measurements. At 1–3 months, record adverse events, treatment changes, and actual compression use. At 6, 12, and 24 months, assess a clearly defined limb-volume measure and patient-reported outcomes, and record prescribed and actual compression use, infection episodes, adverse events, and repeat interventions.	Blind assessors of objective outcomes where possible. Use responder analyses only when the threshold is justified for the scale and context.	Clinical effectiveness outcomes. Their hierarchy is defined in the protocol for each study.	Validated PROMs and objective methods are available. A threshold for within-person change and a criterion for the clinical importance of a between-group difference are not interchangeable. Their applicability depends on the scale and context.

The minimum clinical dataset and assessment schedule are shown in Figure 2. Additional measurements of anastomosis status, transport, and tissue characteristics are optional and can be selected according to the study question and available resources. The proposed timing can be adapted to the study question.

Table S7. Existing clinical and research frameworks and the proposed addition.

Existing framework	What it includes	Purpose	Limitations for the questions addressed in this review	What the proposed sequential measurement framework adds
ISL clinical staging (2023) [32]	Pitting, reversibility or persistence of edema, and tissue and trophic changes	Clinical stage and general description of disease severity	It does not quantitatively measure local function of the selected lymphatic collector, transport through LVA, or tissue composition. Stage is not a validated rule for predicting benefit from LVA.	Retains ISL stage as a baseline clinical descriptor and distinguishes it from optional measurements of anastomosis status, transport, and tissue characteristics.
ICG staging of dermal backflow, such as Narushima et al. (2016) [20]	Superficial fluorescence patterns, linear lymphatic collectors, and dermal backflow	Superficial mapping and characterization of the imaging phenotype	Several nonidentical systems exist. ICG does not measure tissue composition or overall transport reserve and has not been validated as an individual predictor of the comparative benefit of LVA.	Requires the staging system to be specified. ICG is used as a separate imaging measure and does not replace subsequent assessment of transport or patency.
Intraoperative NECST morphological classification (Mihara et al., 2012) [30]	Macroscopic morphology of an individual lymphatic collector, with described histological correlates	Standardized description of the donor lymphatic collector	Developed in lower-extremity lymphedema after treatment for gynecologic cancers. It applies to an individual vessel and has not been validated as a predictor of clinical response in BCRL.	Use as a candidate descriptor of the donor vessel rather than as a whole-limb stage or a stand-alone selection rule. Assess its functional role separately.
BSO classification of the recipient venule (Bianchi et al., 2022, with the hydropower extension by Visconti, 2024) [31,33]	Observed intraoperative dynamics of the recipient venule	Standardization of visual descriptions of the venous side of LVA	Derived from a mixed, nonconsecutive cohort. The visual phenotype is not a direct measurement of pressure or the pressure gradient and has not undergone external prognostic validation.	Record direction or reflux, caliber, valves, limb position, and, where feasible, pressure separately. Analyze proximal venous outflow separately.
Surgical selection algorithm for BCRL (Lilja et al., 2024) [11]	Clinical phenotype, ICG-visible lymphatic collectors, and presumed suitability for different procedures	A structured approach to choosing among LVA, VLNT, and reductive strategies	Based predominantly on observational studies with a high risk of bias. It is not a validated rule for selecting patients expected to derive greater treatment benefit.	Does not compete with the selection algorithm. The framework describes what to measure after a strategy has been chosen and allows separate assessment of technical suitability, prognosis, and possible treatment effect modification.
Expert consensus statements (Sacks et al., 2024, and Masià et al., 2026) [34,35]	Candidacy, preoperative preparation, technique or strategy, and postoperative management	Standardization of clinical practice and expert decisions	Consensus describes practice but does not, by itself, measure the comparative treatment effect or mechanism.	Adds research roles and a temporal ordering of variables while retaining expert recommendations as clinical context.

Existing framework	What it includes	Purpose	Limitations for the questions addressed in this review	What the proposed sequential measurement framework adds
BCRL core outcome set and core outcome measures (Doubblestein et al., 2024) [36,37]	12 outcome domains with recommendations that account for the BCRL phase and available time and resources. Measurement instruments are considered in a separate related publication.	Standardization of patient-important and clinical outcomes in BCRL	They are not specific to LVA delivery and do not describe technical completion, patency, or directional transport through the anastomosis.	Uses these recommendations to inform the clinical dataset and coordinates assessment times, compression documentation, and the procedure record. Additional measurements of anastomosis status, transport, and tissue characteristics are optional.
Society of Lymphatic Surgery minimum outcome standard for lymphatic surgery (Fanning et al., 2025) [38]	Compression, limb volume, PROMs, cellulitis, follow-up duration, and surveillance parameters	Minimum standardization of outcome reporting in lymphatic surgery	No consensus was reached on measuring time spent in compression or the L-Dex threshold. Functional delivery through an individual LVA is not part of the minimum set.	Coordinates the clinical outcomes with assessment times and documentation of prescribed and actual compression use. Records the procedure performed in patients undergoing LVA; additional assessment of patency and transport is optional.
General principles for distinguishing treatment effect, mechanism, and prognosis	Comparative designs, postrandomization variables, and prognostic models	The rationale for interpreting different research questions	They are not specific to lymphatic surgery and do not specify which LVA measures should be collected.	Maps variables used in LVA studies to their analytical roles and specifies their combined measurement in sequence.

Supplemental Methods S1. Treatment effect, prognosis, and investigation of mechanism

The following methodological considerations for future studies of therapeutic LVA in BCRL build on the sequential measurement framework proposed in this review. This section does not describe statistical analyses performed by the authors and is not a clinical trial protocol. The choice of specific methods depends on the research question, available measurements, and adequacy of the data.

S1.1. Overall clinical treatment effect

Before the study begins, define the target population, treatment strategies to be compared, primary outcome, assessment time point, and how the between-group difference will be expressed. The primary outcome should match the research question. PROMs, volume, infection episodes, and compression burden do not form a universal hierarchy. Specify separately how to handle switching to another strategy, repeat operations, changes in concomitant therapy, and discontinuation of follow-up.

To estimate the effect of assignment to a strategy, the primary randomized analysis retains participants in their originally assigned groups according to the intention-to-treat principle. Technical failure, lack of confirmed patency, or refusal of surgery are not grounds for excluding a participant. Postoperative compression, LVA completion, and functional measures should not automatically be entered as ordinary baseline covariates, because doing so may change the treatment effect being estimated and introduce bias. Specify permissible adjustment for baseline characteristics in advance. An analysis of adherence to the strategy requires a separate definition of the target estimand and assumptions. Nonrandomized comparisons may target the same treatment effect but require adequate measurement of factors influencing treatment selection and justification of confounding control.

S1.2. Temporal sequence and measurement quality

Measure baseline clinical and tissue characteristics before the intervention. For prognostic analyses, prespecify the time at which the prediction is made. All predictors must be available by that time, and the predicted outcome must be assessed later. To study a sequence from functional change to tissue change to clinical outcome, schedule assessments at separate time points. Simultaneous measurements can describe associations but cannot establish the order of changes.

Record technical completion, directional transport, patency, and quantitative local flow separately. Include quantitative measurements only when the method is suitable for the specific anatomical site and time point. Do not assume in advance that reproducible longitudinal flowmetry is available. Standardize limb position, physical activity, time of day, the interval after compression removal, and equipment settings. Assess repeatability, agreement between operators, and measurement error before analysis. Interpret differences in the prognostic value of measures in light of their differing precision.

S1.3. Compression and causal analysis of mechanism

Record baseline compression, postoperative prescription, actual hours of wear, garment type, and the dates and reasons for reducing, discontinuing, and resuming use separately. Depending on the prespecified question, these data may characterize concomitant treatment, an outcome, or a hypothesized mediator. Patient-initiated and protocol-directed reductions in compression should not be treated as equivalent interventions.

Randomization of treatment does not eliminate confounding between a postoperative intermediate measure and the clinical outcome. Compression may change in response to surgery and the preceding condition of the limb while also affecting subsequent functional and clinical measures. Ordinary regression adjustment for postoperative compression therefore does not guarantee a valid estimate of mediation. Causal analysis requires specification of the target direct or indirect effect, temporal structure, measured confounders, necessary assumptions, and sensitivity analyses. A specialized longitudinal method may be needed when confounding is affected by prior treatment. Flow through a surgical anastomosis is not comparably defined in patients who have not undergone surgery. Assigning a value of zero to control patients does not resolve this problem. AGReMA provides guidance for transparent reporting but does not, by itself, ensure causal identification. [39]

S1.4. Three prognostic hypotheses

The first hypothesis is that an early functional characteristic adds information about a subsequent tissue outcome beyond its baseline value and prespecified clinical characteristics. Compare the base and extended models in the same target population and at the same time point. Do not include predictors measured after the time at which the prediction is made.

The second hypothesis is that the additional information provided by a functional measure differs for a fluid-sensitive outcome and total limb volume. Assess both outcomes at the same follow-up time point while accounting for their baseline values. Prespecify a comparable dimensionless measure for the comparison, such as the relative reduction in prediction error compared with the corresponding base model. Errors expressed in milliliters and BIS units cannot be compared directly. Estimate the difference in additional information itself and its uncertainty. A significant result for one outcome and a nonsignificant result for the other does not establish a difference between the associations.

The third hypothesis is that the prognostic association between a functional characteristic and the outcome depends on baseline tissue phenotype. Test a prespecified interaction among surgical patients and avoid choosing thresholds after reviewing the results. Such an interaction is not modification of the comparative treatment effect. The latter requires comparison of treatment strategies and characteristics measured comparably before treatment in both groups. Intraoperative NECST alone does not meet this requirement.

S1.5. Unit of analysis, denominators, and missing data

For clinical prognosis, the patient is the unit of analysis. When there are multiple anastomoses, prespecify how to derive a patient-level measure or use a model that accounts for dependence among measurements. Individual anastomoses do not increase the number of independent patients. Flow rates may be summed only under comparable conditions and without double-counting fluid in successive segments of the same pathway. Split data for model validation and perform resampling at the patient level, keeping all anastomoses and visits for each patient together.

At each time point, report the numbers of patients and anastomoses due for assessment and actually examined, the actual denominators, and reasons for missing data. Distinguish confirmed patency, confirmed occlusion, an indeterminate assessment, an examination not performed, and loss to follow-up as specified in Table S3. A missing measurement or uninformative imaging result is not equivalent to zero flow. Distinguish structural inapplicability, such as flow through an anastomosis that was never created, from a missing measurement. For genuinely missing data, prespecify assumptions and handling methods, then assess whether conclusions hold under plausible departures from those assumptions. Complete-case analysis requires justification.

S1.6. Sample size, model validation, and multiplicity

Plan sample sizes separately for the clinical comparison, prognostic model development, and interaction testing. For a model, account for the number of parameters to be estimated, the type and distribution of the outcome, expected prognostic information, overfitting, and data loss. A universal rule for the number of patients per predictor does not replace a sample size calculation. The external validation sample must be large enough to estimate model performance with adequate precision. [40]

Conduct internal validation using resampling or cross-validation, incorporating all steps of feature selection, data transformation, missing-data handling, and model tuning. Report prediction error, calibration, and, where applicable, discrimination, with estimates of uncertainty. External validation in an independent population is required before clinical use. TRIPOD+AI provides reporting guidance for clinical prediction models that use regression or machine learning methods. [41]

Prespecify the primary outcome, time point, confirmatory hypotheses, and approach to multiple comparisons. Clearly label analyses of additional domains, time points, and interactions as confirmatory or exploratory. Failure to detect a prognostic association may reflect inadequate precision, a narrow range of the measure, measurement error, or a lack of useful additional information. Biological plausibility does not justify including a measure without demonstrated prognostic value. At the same time, failure of prediction does not disprove all possible mechanisms of LVA.

References

1. Jonis, Y.M.J.; Wolfs, J.A.G.N.; Hummelink, S.; Tielemans, H.J.P.; Keuter, X.H.A.; van Kuijk, S.; Ulrich, D.J.O.; van der Hulst, R.R.W.J.; Qiu, S.S. The 6 month interim analysis of a randomized controlled trial assessing the quality of life in patients with breast cancer related lymphedema undergoing lymphaticovenous anastomosis vs. conservative therapy. *Scientific Reports* **2024**, *14*, 2238, doi:10.1038/s41598-024-52489-3.
2. Kleeven, A.; Jonis, Y.M.J.; Currie, O.; Wolfs, J.; Kimman, M.; Tielemans, H.; van der Hulst, R.R.W.J.; Hummelink, S.; Qiu, S.S. Economic evaluation of lymphaticovenous anastomosis versus conservative therapy for breast cancer-related lymphoedema: secondary outcome analysis of a randomized clinical trial. *British Journal of Surgery* **2026**, *113*, znag062, doi:10.1093/bjs/znag062.
3. Mihara, M.; Hara, H.; Kawasaki, Y.; Mitsuhashi, T.; Orikasa, H.; Ando, H.; Naito, M. Lymphatic venous anastomosis and complex decongestive therapy for lymphoedema: randomized clinical trial. *British Journal of Surgery* **2024**, *111*, znad372, doi:10.1093/bjs/znad372.
4. Shimbo, K.; Kawamoto, H.; Koshima, I. Comparative study of conservative treatment and lymphaticovenular anastomosis with compression therapy for early-stage breast cancer-related lymphoedema. *Journal of Plastic, Reconstructive & Aesthetic Surgery* **2024**, *88*, 390-396, doi:10.1016/j.bjps.2023.11.035.
5. Seidenstuecker, K.; Fertsch, S.; Ghazaleh, A.A.; Fabi, A.; Stoffel, J.; Bukowiecki, J.; Wolter, A.; Aghlmandi, S.; Nadella, A.; Halbeisen, F.S.; et al. Improving quality of life after breast cancer: a comparison of two microsurgical treatment options for breast cancer-related lymphedema (BCRL). *Clinical and Experimental Medicine* **2024**, *24*, 82, doi:10.1007/s10238-024-01344-w.
6. Kappos, E.A.; Fabi, A.; Halbeisen, F.S.; Abu-Ghazaleh, A.; Stoffel, J.; Aufmesser-Freyhardt, B.; Bukowiecki, J.; Handschin, T.M.; Andree, C.; Haug, M.D.; et al. Vascularized lymph node transfer (VLNT) versus lymphaticovenous anastomosis (LVA) for chronic breast cancer-related lymphedema (BCRL): a retrospective cohort study of effectiveness over time. *Breast Cancer Research and Treatment* **2025**, *210*, 319-327, doi:10.1007/s10549-024-07567-5.
7. Zeltzer, A.A.; Nistor, A.; Adriaenssens, N.; Deleuze, J.; Giunta, G.; Hamdi, M. Outcomes of Lymphovenous Anastomoses and Vascularized Lymph Node Transplant in the Combined Surgical Treatment of Lymphedema: A Prospective Cohort Study. *Plastic and reconstructive surgery* **2026**, *157*, 163-175, doi:10.1097/PRS.0000000000012326.
8. Rosian, K.; Stanak, M. Efficacy and safety assessment of lymphovenous anastomosis in patients with primary and secondary lymphoedema: A systematic review of prospective evidence. *Microsurgery* **2019**, *39*, 763-772, doi:10.1002/micr.30514.
9. Chang, D.W.; Dayan, J.; Greene, A.K.; MacDonald, J.K.; Masia, J.; Mehrara, B.; Neligan, P.C.; Nguyen, D. Surgical Treatment of Lymphedema: A Systematic Review and Meta-Analysis of Controlled Trials. Results of a Consensus Conference. *Plastic & Reconstructive Surgery* **2021**, *147*, 975-993, doi:10.1097/PRS.0000000000007783.
10. Meuli, J.N.; Guiotto, M.; Elmers, J.; Mazzolai, L.; di Summa, P.G. Outcomes after microsurgical treatment of lymphedema: a systematic review and meta-analysis. *International Journal of Surgery* **2023**, *109*, 1360-1372, doi:10.1097/JS9.0000000000000210.
11. Lilja, C.; Madsen, C.B.; Damsgaard, T.E.; Sørensen, J.A.; Thomsen, J.B. Surgical treatment algorithm for breast cancer lymphedema-a systematic review. *Gland Surgery* **2024**, *13*, 722-748, doi:10.21037/gs-23-503.
12. Zapata-Ospina, A.; Lopera-Muñetón, C.; Betancur-Bedoya, S.P.; Ángel-Bustos, I.C.; Vásquez-Montoya, M.G. Effectiveness of Lymphovenular Anastomosis and Complex Decongestive Therapy for the Treatment of Lymphedema in Patients with Breast Cancer: A Systematic Review. *Lymphatic Research and Biology* **2024**, *22*, 232-240, doi:10.1089/lrb.2024.0014.
13. Hahn, B.A.; Kleeven, A.; Richir, M.C.; Witkamp, A.J.; Kuijpers, A.M.J.; de Jong, T.; Qiu, S.; Coert, J.H.; Krijgh, D.D. Objectifying Clinical Outcomes After Lymphaticovenous Anastomosis and Vascularized Lymph Node Transfer in the Treatment of Extremity Lymphedema: A Systematic Review and Meta-Analysis. *Microsurgery* **2025**, *45*, e70050, doi:10.1002/micr.70050.
14. Zurfluh, C.; Ullmann, H.; Tung, W.S.; Grünherz, L.; Harder, Y.; Giovanoli, P.; Lindenblatt, N. Beyond volume reduction: Systematic review and meta-analysis of microsurgical treatment of lymphedema. *Journal of Plastic, Reconstructive & Aesthetic Surgery* **2025**, *111*, 272-284, doi:10.1016/j.bjps.2025.10.028.
15. Nicholas Jungbauer, W., Jr.; Solomon, S.; Verhey, E.M.; Jeger, J.L.; Chang, Y.H.; Rhee, D.H.; Rebecca, A.M.; Saint-Cyr, M.; Reece, E.M.; Casey, W.J., 3rd. Lymphovenous Anastomosis and Vascularized Lymph Node Transfer Reduce Long-term Cellulitis Events in Patients With Secondary Lymphedema: A Systematic Review and Meta-analysis. *Annals of Plastic Surgery* **2025**, *95*, 522-530, doi:10.1097/SAP.0000000000004508.
16. Brown, S.; Shen, Y.; Klimitz, F.J.; Nair, M.; Glaeser-Khan, S.; Shamoun, V.; Pomahac, B.; Lotfi, P.; Golshan, M.; Haykal, S. Lymphovenous bypass for the treatment of secondary lymphedema: A meta-analysis of prospective outcomes. *Breast Cancer Research and Treatment* **2026**, *215*, 42, doi:10.1007/s10549-025-07867-4.
17. Kumar, S.I.S.; Sousa, B.A.; de Luna Barros, M.M.; Katta, T.; Garcia, J.; Moreira, A.; Butterworth, J. Quality-of-life impact of lymphovenous anastomosis in breast cancer-related lymphedema: A systematic review and

meta-analysis. *Journal of Plastic, Reconstructive & Aesthetic Surgery* **2026**, *117*, 146-159, doi:10.1016/j.bjps.2026.03.046.
18. Zhang, C.; Lodi, M. Immediate and delayed lymphatic reconstruction of breast-cancer related lymphedema: A systematic review and network meta-analysis. *European Journal of Surgical Oncology* **2026**, *52*, 111394, doi:10.1016/j.ejso.2026.111394.
19. Gong, X.; Wang, L.; Liu, F.; Zhang, J.; Xiao, H.; Hou, Y.; Guo, X.; Liu, Z. The application of contrast-enhanced ultrasound combined with indocyanine green lymphography in the management of secondary upper limb lymphedema. *Cancer Research and Treatment* **2025**, doi:10.4143/crt.2025.813.
20. Narushima, M.; Yamamoto, T.; Ogata, F.; Yoshimatsu, H.; Mihara, M.; Koshima, I. Indocyanine Green Lymphography Findings in Limb Lymphedema. *Journal of reconstructive microsurgery* **2016**, *32*, 72-79, doi:10.1055/s-0035-1564608.
21. Hashem, T.; Yamamoto, T. Supermicrosurgical lymphaticovenous anastomosis for breast cancer related lymphedema in low resources settings. *World Journal of Surgical Oncology* **2025**, *23*, 156, doi:10.1186/s12957-025-03756-8.
22. Kim, C.; Lee, C.; Queiros, I.; Mercado, N.; Fanning, J.E.; Weinstein, J.; Singhal, D.; Tsai, L.L. Noncontrast ultra-high-frequency ultrasound for preoperative lymphovenous mapping in patients undergoing lymphaticovenous anastomosis surgery for extremity lymphedema. *AJR American Journal of Roentgenology* **2025**, *224*, e2432001, doi:10.2214/AJR.24.32001.
23. Tang, Y.; Huang, X.; Liang, Z.; Yu, X.; Liu, Z.; Zhu, J. Contrast-enhanced ultrasound combined with ultra-high-frequency ultrasound improves preoperative planning for lymphovenous anastomosis: A pilot study. *Journal of vascular surgery. Venous and lymphatic disorders* **2026**, *14*, 102361, doi:10.1016/j.jvsv.2025.102361.
24. Miseré, R.M.L.; Qiu, S.S.; Ewalds, L.; van der Hulst, R.R.W.J. Lymph flow before and after lymphaticovenous anastomosis measured using Transonic transit-time ultrasound microvascular flowprobe. *Lymphatic Research and Biology* **2021**, *19*, 539-544, doi:10.1089/lrb.2019.0088.
25. Zhu, J.; Guo, Q.; Huang, Y.; Zhu, Y.; Hou, H.; Wang, N.; Ren, H.; Yu, X.; Mao, G.; Lin, B.; et al. Clinical value of contrast-enhanced magnetic resonance lymphatic vessel imaging for precise localization of lymphatic vessels in patients with lymphaticovenous anastomosis. *Journal of plastic, reconstructive & aesthetic surgery : JPRAS* **2025**, *105*, 385-394, doi:10.1016/j.bjps.2025.04.017.
26. He, H.; Yu, J.; Xiang, Y.; Luo, T.; Tao, S. Accuracy of shear-wave elastography in diagnosing severity of breast cancer-related lymphedema. *Ultrasound* **2026**, doi:10.1177/1742271X261446207.
27. Ridner, S.H.; Dietrich, M.S.; Boyages, J.; Koelmeyer, L.; Elder, E.; Hughes, T.M.; French, J.; Ngui, N.; Hsu, J.; Abramson, V.G.; et al. A comparison of bioimpedance spectroscopy or tape measure triggered compression intervention in chronic breast cancer lymphedema prevention. *Lymphatic Research and Biology* **2022**, *20*, 618-628, doi:10.1089/lrb.2021.0084.
28. Li, Y.; Tang, J.; Mao, D.; Dragomir, M.P.; Li, Y.; Sun, K.; Lv, Z.; Liu, X.; Meng, X.; Lu, K. MRI-CEUS fusion-guided lymphatic mapping as a preoperative strategy for lymphedema patients undergoing lymphaticovenous anastomosis surgery. *Journal of Vascular Surgery: Venous and Lymphatic Disorders* **2024**, *12*, 101907, doi:10.1016/j.jvsv.2024.101907.
29. Suzuki, Y.; Kajita, H.; Otaki, M.; Watanabe, S.; Nagashima, H.; Okabe, K.; Imanishi, N.; Sakuma, H.; Kishi, K. Evaluation of postoperative anastomotic patency in lymphaticovenular anastomosis using photoacoustic imaging. *Journal of Surgical Oncology* **2025**, *131*, 42-46, doi:10.1002/jso.27898.
30. Mihara, M.; Hara, H.; Hayashi, Y.; Narushima, M.; Yamamoto, T.; Todokoro, T.; Iida, T.; Sawamoto, N.; Araki, J.; Kikuchi, K.; et al. Pathological steps of cancer-related lymphedema: histological changes in the collecting lymphatic vessels after lymphadenectomy. *PLoS One* **2012**, *7*, e41126, doi:10.1371/journal.pone.0041126.
31. Bianchi, A.; Salgarello, M.; Hayashi, A.; Yang, J.C.; Visconti, G. Recipient Venule Selection and Anastomosis Configuration for Lymphaticovenular Anastomosis in Extremity Lymphedema: Algorithm Based on 1,000 Lymphaticovenular Anastomosis. *Journal of reconstructive microsurgery* **2022**, *38*, 472-480, doi:10.1055/s-0041-1735836.
32. Executive Committee of the International Society of Lymphology. The Diagnosis and Treatment of Peripheral Lymphedema: 2023 Consensus Document of The International Society of Lymphology. *Lymphology* **2023**, *56*, 133-151, doi:10.2458/lymph.6372.
33. Visconti, G. The Recipient Venule in LVA. In *Supermicrosurgical Lymphaticovenular Anastomosis*, Visconti, G., Hayashi, A., Yang, J.C.S., Eds.; Springer: Cham, 2024; pp. 115-132.
34. Sacks, J.; Riley, B.; Doubblestein, D.; Kirby, J.P.; Towers, A.; Weatherly, K. Expert-consensus on lymphedema surgeries: candidacy, prehabilitation, and postoperative care. *Medical Oncology* **2024**, *41*, 266, doi:10.1007/s12032-024-02449-8.
35. Masià, J.; Al Sakkaf, A.M.; Hong, J.P.; Neligan, P.; Pons, G.; Rovnaya, A.; Martínez-Jaimez, P. Barcelona Consensus Paper on Reconstructive Lymphedema Surgery: A Delphi Study. *Plastic & Reconstructive Surgery* **2026**, *157*, 193-203, doi:10.1097/PRS.0000000000012201.
36. Doubblestein, D.; Koehler, L.; Anderson, E.; Scheiman, N.; Stewart, P.; Schaverien, M.; Armer, J. Development of a core outcome set for breast cancer-related lymphedema: a Delphi study. *Breast Cancer Research and Treatment* **2024**, *205*, 359-370, doi:10.1007/s10549-024-07262-5.

37. Doubblestein, D.; Koehler, L.; Anderson, E.; Scheiman, N.; Stewart, P.; Schaverien, M.; Armer, J. Development of a core set of outcome measures to be applied toward breast cancer-related lymphedema core outcome domains. *Breast Cancer Research and Treatment* **2024**, *205*, 439-449, doi:10.1007/s10549-024-07298-7.
38. Fanning, J.E.; Aly, M.A.I.; Chang, D.W.; Dayan, J.H.; Higgins, J.P.; Lee, B.T.; Masià, J.; Mehrara, B.J.; Neligan, P.C.; Smith, M.L.; et al. A Proposed Minimum Standard Set of Outcome Measures for Lymphatic Surgery: Results of a Modified Delphi Process from the Society of Lymphatic Surgery Leadership. *Plastic & Reconstructive Surgery* **2025**, doi:10.1097/PRS.0000000000012793.
39. Lee, H.; Cashin, A.G.; Lamb, S.E.; Hopewell, S.; Vansteelandt, S.; VanderWeele, T.J.; MacKinnon, D.P.; Mansell, G.; Collins, G.S.; Golub, R.M.; et al. A Guideline for Reporting Mediation Analyses of Randomized Trials and Observational Studies: The AGReMA Statement. *JAMA* **2021**, *326*, 1045-1056, doi:10.1001/jama.2021.14075.
40. Riley, R.D.; Ensor, J.; Snell, K.I.E.; Harrell, F.E., Jr; Martin, G.P.; Reitsma, J.B.; Moons, K.G.M.; Collins, G.; van Smeden, M. Calculating the sample size required for developing a clinical prediction model. *BMJ* **2020**, *368*, m441, doi:10.1136/bmj.m441.
41. Collins, G.S.; Moons, K.G.M.; Dhiman, P.; Riley, R.D.; Beam, A.L.; Van Calster, B.; Ghassemi, M.; Liu, X.; Reitsma, J.B.; van Smeden, M.; et al. TRIPOD+AI statement: updated guidance for reporting clinical prediction models that use regression or machine learning methods. *BMJ* **2024**, *385*, e078378, doi:10.1136/bmj-2023-078378.
